# Comprehensive bioinformatics analysis reveals the prognostic value, predictive value, and immunological roles of ANLN in human cancers

**DOI:** 10.3389/fgene.2022.1000339

**Published:** 2022-09-20

**Authors:** Zhiwei Cui, Jiantao Mo, Ping Song, Lijun Wang, Rongli Wang, Feiyan Cheng, Lihui Wang, Fan Zou, Xin Guan, Nini Zheng, Xinyuan Yang, Wei Wang

**Affiliations:** ^1^ Department of Obstetrics and Gynecology, The First Affiliated Hospital of Xi’an Jiaotong University, Xi’an, China; ^2^ Department of Hepatobiliary Surgery, The First Affiliated Hospital of Xi’an Jiaotong University, Xi’an, China; ^3^ Department of Gastroenterology, The Second Affiliated Hospital of Xi’an Jiaotong University, Xi’an, China; ^4^ Department of Respiratory and Critical Care Medicine, Affiliated Hospital of Zunyi Medical University, Zunyi, China; ^5^ Department of Anesthesiology, The First Affiliated Hospital of Xi’an Jiaotong University, Xi’an, China

**Keywords:** ANLN, cell cycle, mitosis, tumor immunity, p53 signaling

## Abstract

Anillin (ANLN) is a unique scaffolding, actin-binding protein, which is essential for the integrity and ingression of the cleavage furrow. It is mainly involved in the cytokinesis process, while its role in various tumors has not been fully addressed and remains largely elusive. To provide a thorough perspective of ANLN’s roles among diverse malignancies, we conducted a comprehensive, pan-cancer analysis about ANLN, including but not limited to gene expression levels, prognostic value, biological functions, interacting proteins, immune-related analysis, and predictive value. As a result, when compared to normal tissues, ANLN expression is elevated in most cancers, and its expression also differs in different immune subtypes and molecular subtypes in diverse cancers. In addition, in 17 types of cancer, ANLN expression is increased in early tumor stages, and higher ANLN expression predicts worse survival outcomes in more than ten cancers. Furthermore, ANLN shows close correlations with the infiltration levels of most immune cells, and enrichment analysis using ANLN co-expressed genes reveals that ANLN plays essential roles in cell cycle, mitosis, cellular senescence, and p53 signaling pathways. In the final, ANLN exhibits high accuracy in predicting many cancers, and subsequent multivariate analysis suggests ANLN could be an independent prognostic factor in specific cancer types. Taken together, ANLN is proved to be a novel and promising biomarker for its excellent predictive utility, promising prognostic value, and potential immunological roles in pan-cancer. Targeting ANLN might be an attractive approach to tumor treatment.

## Introduction

Anillin is an evolutionarily conserved actin-binding protein, and it is first identified in Drosophila. The ANLN gene, which is found on chromosome 7p14.2, codes for a cytoskeletal scaffolding protein of 1125 amino acids, which plays crucial roles in the maintenance of appropriate cytokinetic furrow positioning and the formation of stable midbody in the process of cytokinesis (Wang et al., 2019). The deficiency of ANLN results in the slowdown of the ingression and cytokinesis failure (Kučera et al., 2021). Anillin homology (AH) and pleckstrin homology (PH) domains are found at the C-terminus of ANLN. The former domain binds RhoA, and the latter is crucial for Anillin recruitment to the equatorial membrane (Kim et al., 2017). ANLN regulates cell contractility through binding to GTP-RhoA, F-actin, activated non-muscle Myosin II (NMII), and other cytoskeletal regulators, like mDia1 and septins (Morris et al., 2020). Apart from its previously described function as organizing and stabilizing actomyosin contractile rings in cytokinesis, recent studies have unveiled its important role in maintaining cell-cell junctions and integrity, as well as regulating cell migration, through adjusting the distribution of Rho-GTP and stabilizing actin filaments, respectively (Reyes et al., 2014; Tian et al., 2015). In addition, ANLN also functions as a scaffolding molecule to promote cellular interactions and signaling pathways (Morris et al., 2020). The localization of ANLN in the cell cycle is not constant. ANLN has dynamic intracellular localization, shuttling between the nucleus and cytoplasm. It dominantly localizes to the nucleus in interphase. However, it will re-localize evenly in the cell cortex upon entering into mitosis. In the late mitotic phase, before the commencement of cytokinesis, ANLN departs from the poles and accumulates in the equatorial zone (Kim et al., 2017).

As a critical regulator of cell division, cell junction, and cytokinesis, it is not surprising that ANLN is closely associated with tumor initiation and progression. Previous research has demonstrated that ANLN mRNA expression is upregulated in cancerous tumors by 2 to 6 fold, which is higher than the fold of Ki-67, a famous tumor proliferative nuclear marker (Hall et al., 2005; Menon et al., 2019). In addition, human tumor metastatic and progressive potential is closely associated with the expression levels of ANLN (Zhang and Maddox, 2010). There is growing evidence linking ANLN to the development of different types of tumors. Worldwide, breast cancer, an obesity-related malignancy, is still the most prevalent cancer (Loibl et al., 2021; Chen et al., 2022). ANLN is reported to boost breast cancer cell growth, migration, metastasis, and drug resistance (Zhou et al., 2015; Wang D. et al., 2020; Wang F. et al., 2020). New lung cancer cases per year are estimated to be 2 million worldwide, making lung cancer one of the most deadly cancers (Thai et al., 2021). In lung adenocarcinoma, ANLN is identified as a potential prognostic marker and may affect the epithelial-mesenchymal transition process (Long et al., 2018; Xu et al., 2019). Furthermore, there is still significant morbidity and mortality associated with pancreatic cancer, one of the deadliest types of cancer (Mizrahi et al., 2020). It is reported that ANLN participates in the HMGA2-induced increase in the tumorigenicity of pancreatic cancer cells and through controlling the EZH2/miR-218-5p/LASP1 axis, ANLN deficiency dramatically reduces pancreatic tumor cell migration and invasion (Wang et al., 2019; Guo et al., 2020). Consequently, ANLN appears to be a promising prognostic biomarker and an intriguing therapeutic target for the accurate diagnosis and precise treatment of tumor patients. Nevertheless, current research merely focuses on fixed types of cancer, and the potential effect of ANLN on commonly diagnosed gynecological tumors, like endometrial cancer, and malignant kidney tumors, which are characterized by multiple histological subtypes, is still unclear (Turajlic et al., 2018; Ni et al., 2022). Therefore, it is necessary to analyze the role of ANLN from a pan-cancer perspective.

The current research first explored ANLN expression in pan-cancer and identified that both mRNA and protein levels of ANLN expression were upregulated in most tumor tissues compared to normal tissues. In addition, ANLN expression increased in early tumor stages in most cancers. We next revealed that ANLN had exceptional and robust predictive value in predicting more than ten cancer types, including breast invasive carcinoma (BRCA), cervical squamous cell carcinoma and endocervical adenocarcinoma (CESC), cholangiocarcinoma (CHOL), colon carcinoma (COAD), esophageal carcinoma (ESCA), and kidney renal clear cell carcinoma (KIRC), as well as prognostic value in several malignancies, including adrenocortical carcinoma (ACC), bladder urothelial carcinoma (BLCA), BRCA, CESC, lung adenocarcinoma (LUAD), liver hepatocellular carcinoma (LIHC), and kidney cancer. Moreover, ANLN co-expressed genes were predominantly involved in many cell cycle and DNA replication-related pathways. Furthermore, we also found that ANLN expression was linked to the infiltration levels of many immune cells, and it could predict the immune checkpoint blockade (ICB) response in specific cancers. Final Cox regression analyses unveiled that ANLN could serve as an independent prognostic biomarker for certain cancers. To conclude, this pan-cancer analysis shed light on the pivotal carcinogenic roles of ANLN and paved the way for future ANLN research in solid tumors.

## Materials and methods

### The collection of expression and survival data of ANLN

The RNA-sequencing (RNA-seq) data and accompanied clinical data of the pan-cancer cohort (*n* = 15,776), including 33 different cancer types derived from The Cancer Genome Atlas (TCGA) and normal tissues of the Genotype-Tissue Expression (GTEx), were downloaded from UCSC XENA. Expression profile data in Transcripts Per Million (TPM) format were log2 transformed and incorporated into subsequent analyses. Additionally, we used the expression data of 36 cohorts and survival information of 20 cohorts from Gene Expression Omnibus (GEO) datasets (Supplementary Table S1) to validate the results.

### Survival analysis of ANLN in pan-cancer

The association between ANLN expression and the patient prognosis in each tumor was investigated using the Cox regression model. Patients’ survival information includes overall survival (OS), disease-specific survival (DSS), disease-free interval (DFI), and progression-free interval (PFI). We drew forest plots to display the results using the R package “forest”. We also utilized the PrognoScan database the assess association between ANLN expression and patient survival outcomes. We also verified the link between ANLN expression and patient’s survival outcome involving OS and relapse-free survival (RFS) in the Kaplan-Meier plotter by splitting patients by the best cutoff.

### Utilizing the online database

From HPA (https://www.proteinatlas.org), we obtained immunohistochemical images of 15 kinds of tumor tissues and their corresponding normal tissues in order to analyze the differential expression of ANLN at the protein level.

The “TCGA” and “CPTAC” modules of the UALCAN (http://ualcan.path.uab.edu/index.html) database were utilized to compare the ANLN promotor methylation status and ANLN protein expression in pan-cancer, respectively.

We used the “Similar Genes Detection” module of GEPIA2 (http://gepia2.cancer-pku.cn/#index) and included all TCGA tumor tissues to acquire the top 100 genes co-expressed with ANLN. We collected these genes and incorporated them into the subsequent enrichment analysis.

The associations between ANLN expression and subtypes across human cancers were performed in the “Subtype” module of the TISIDB (http://cis.hku.hk/TISIDB/index.php) database.

### Functional and pathways enrichment analyses

The 100 genes obtained from GEPIA2 before were brought into function annotations, including BP (biological process), CC (cellular component), MF (molecular function), and KEGG (Kyoto Encyclopedia of Genes and Genomes) using the R package “ClusterProfiler”. We selected the top five results for each item and displayed them with bubble charts. Functions and pathways that differentially existed in high and low expression groups of ANLN in different cancer cohorts were elucidated using gene set enrichment analysis (GSEA), with gene set of “c2. cp.v7.2. symbols.gmt” from MSigDB, and each analysis procedure repeated 5,000 times. Our ridge plots showed the top 15 “Reactom pathways” and corrected the *p-*values with PH.

GeneMANIA (https://genemania.org/) prediction website offered an approach for predicting gene function from the composite network. We input “ANLN, CKAP2L, KIF23, KIF14, RACGAP1, and DEPDC1” and built a functional protein-protein interaction network.

### Association between ANLN and tumor immunity

In this study, we used two algorithms named single sample GSEA (ssGSEA) and ESTIMATE to determine whether ANLN expression correlated with immune cell infiltration. In the former algorithm, specific markers of immune cells were used as gene sets for the calculation of enrichment scores, revealing the infiltration of immune cells in each sample (Bindea et al., 2013). Built-in markers were available for calculating immune, stroma, and ESTIMATE scores with the ESTIMATE algorithm.

We chose eight genes as immune-checkpoint-related transcripts. Their Spearman’s correlations with ANLN expression in pan-cancer were calculated and displayed. The Tumor Immune Dysfunction and Exclusion (TIDE) algorithm used a set of gene expression markers to assess two mechanisms of tumor immune escape (Jiang et al., 2018). Potential ICB response between ANLN-high and low groups in eight cancer types was predicted with the TIDE algorithm and compared with the Wilcoxon test.

The link between ANLN expression and the immune infiltration levels of cancer-associated fibroblast, myeloid-derived suppressor cells, and T cell NK (Nature killer T cells, NKT cells) in pan-cancer was investigated using the TIMER2.0 (timer.cistrome.org).

### Predictive value of ANLN in pan-cancer

We utilized the “pROC” package to draw receiver operation characteristic (ROC) curves to explore the predictive value of ANLN in TCGA tumor tissues and corresponding normal tissues from GTEx and TCGA. The area value under the ROC curve (AUC) ranged from 0.5 to 1. The ROC’s predictive value increased as it got closer to 1. AUC had low accuracy between 0.5 and 0.7, certain accuracy between 0.7 and 0.9, and high accuracy between 0.9 and 1.0.

### Construction and evaluation of nomograms

We first used univariate and multivariate Cox regression analysis to assess the risk factors influencing patients’ OS. Factors with *p*-values of less than 0.1 were included in the subsequent multivariate Cox analysis. We constructed nomograms based on the parameters included in the multivariate analysis. The concordance index (C-index) was formulated as an assessment for the predictive accuracy of the nomogram, with 1000 as the number of repetitions. Calibrations curves were drawn to compare the fitting between predicted OS and actual OS.

### Statistical analysis

R software v3.6.3 was used for statistical analysis, and the “ggplot2” package was for visualization. The Wilcoxon rank-sum test detected the ANLN expression difference between normal and tumor tissues. Wilcoxon signed-rank test detected the ANLN expression difference between tumor and paired normal tissues. By Spearman’s correlation coefficient, the correlations between ANLN and the values of tumor mutation burden (TMB), microsatellite instability (MSI), mutant-allele tumor heterogeneity (MATH), homologous recombination deficiency (HRD), loss of heterozygosity (LOH), neoantigens (NEO), DNA methylation-based score (DNAss), and RNA expression-based score (RNAss) were calculated. Statistical significance was defined as a *p*-value of less than 0.05.

## Result

### ANLN expression is upregulated in the majority of cancers

**FIGURE 1:**
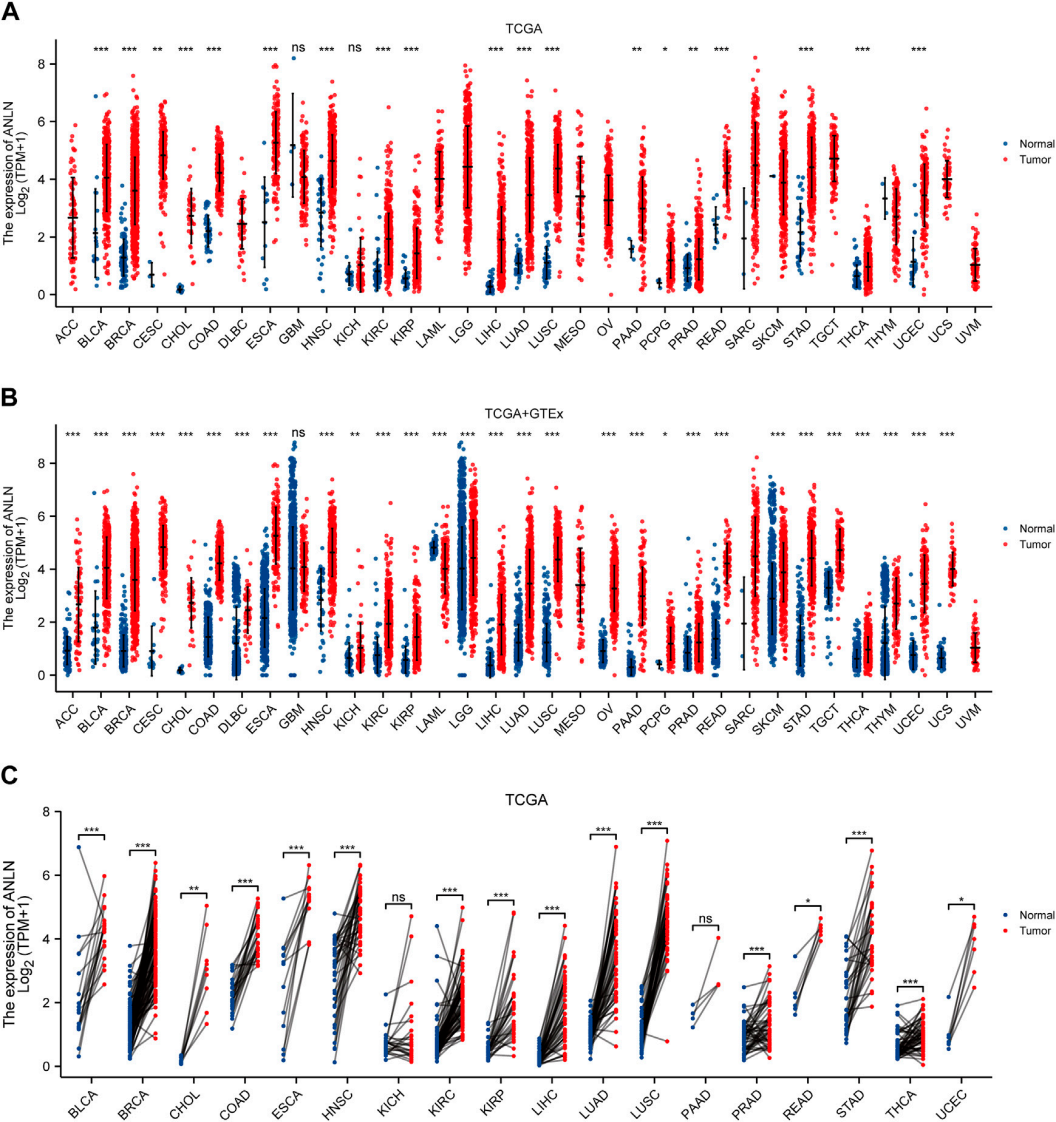
ANLN mRNA expression in tumor and normal tissues. **(A)** ANLN mRNA expression difference between TCGA tumor and normal tissues. **(B)** ANLN mRNA expression difference between tumor and normal tissues with data from the TCGA and GTEx. **(C)** ANLN mRNA expression in TCGA tumor and paired normal tissues (*p < 0.05, **p < 0.01, ***p < 0.001).

We first conducted the expression difference analysis of ANLN mRNA between tumor and normal tissues in the TCGA database. As shown in Figure 1A, ANLN mRNA was substantially elevated in BLCA, BRCA, CESC, CHOL, COAD, ESCA, head, and neck squamous cell carcinoma (HNSC), KIRC, kidney renal papillary cell carcinoma (KIRP), LIHC, LUAD, lung squamous cell carcinoma (LUSC), pancreatic adenocarcinoma (PAAD), pheochromocytoma and paraganglioma (PCPG), prostate adenocarcinoma (PRAD), rectum adenocarcinoma (READ), stomach adenocarcinoma (STAD), thyroid carcinoma (THCA), and uterine corpus endometrial carcinoma (UCEC). No ANLN expression difference was observed in glioblastoma multiforme (GBM) and kidney chromophobe (KICH).

Due to the unavailability and the low number of normal tissues in the TCGA database, we incorporated the GTEx normal tissues and matched them with the TCGA tumor tissues to make the results more convincing. We discovered that ANLN expression was significantly upregulated in 28 cancer types, including ACC, BLCA, BRCA, CESC, CHOL, COAD, lymphoid neoplasm diffuse large B-cell lymphoma (DLBC), ESCA, HNSC, KICH, KIRC, KIRP, brain lower grade glioma (LGG), LIHC, LUAD, LUSC, ovarian serous cystadenocarcinoma (OV), PAAD, PCPG, PRAD, READ, skin cutaneous melanoma (SKCM), STAD, testicular germ cell tumors (TGCT), THCA, thymoma (THYM), UCEC, and uterine carcinosarcoma (UCS). While only in acute myeloid leukemia (LAML), it was significantly downregulated. After we compared ANLN expression among TCGA tumors and adjacent-normal tissues, we observed that among the paired samples from 18 cancers, ANLN mRNA expression was increased in BLCA, BRCA, CHOL, COAD, ESCA, HNSC, KIRC, KIRP, LIHC, LUAD, LUSC, PRAD, READ, STAD, THCA, and UCEC (Figure 1C).

We collected and collated 36 independent cohorts from the GEO database covering more than 20 cancer types to validate our results further. The results consistently indicated that ANLN showed significant and higher expression in tumor tissues (Supplementary Figures S1A–C). We conjured that ANLN was dysregulated and highly expressed during tumor formation.

Next, the protein expression and promoter methylation levels of ANLN were explored by the UALCAN. Promoter methylation levels of ANLN were lower in tumor patients with BLCA, BRCA, HNSC, KIRP, LIHC, LUAD, PRAD, READ, STAD, THYM, THCA, and UCEC. In contrast, patients with LUSC or sarcoma (SARC) showed higher ANLN promoter methylation levels (Supplementary Figures S2A–N). No significant difference was found in CESC, COHL, COAD, ESCA, GBM, KIRC, PAAD, PCPG, and TGCT. We observed the ANLN protein expression levels in ten cancer types and discovered that 9 out of 10 had higher ANLN protein expression than normal tissues, including BRCA, colon cancer, HNSC, KIRC LIHC, LUAD, OV, PAAD, and UCEC. However, patients with GBM tended to have lower ANLN protein expression (Supplementary Figures S3A–J).

**FIGURE 2:**
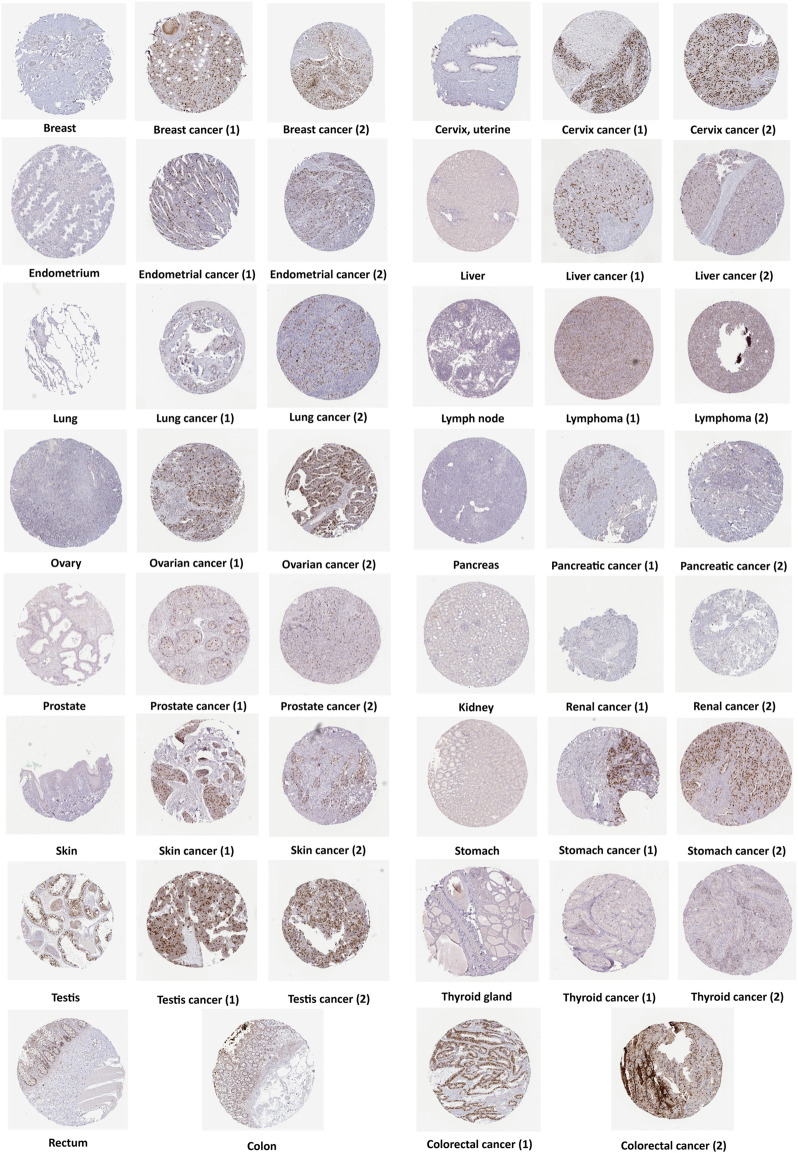
ANLN protein expression in immunohistochemical images of normal (left) and tumor (right) groups.

Moreover, we used the HPA database to elicit immunohistochemical images to determine the protein expression level of ANLN. As can be seen in Figure 2, the protein expression of ANLN was significantly higher in 15 cancers than in normal tissues. To sum, both ANLN mRNA and protein were upregulated in most cancers.

### ANLN expression is negatively correlated with patient prognosis in most cancer types

**FIGURE 3:**
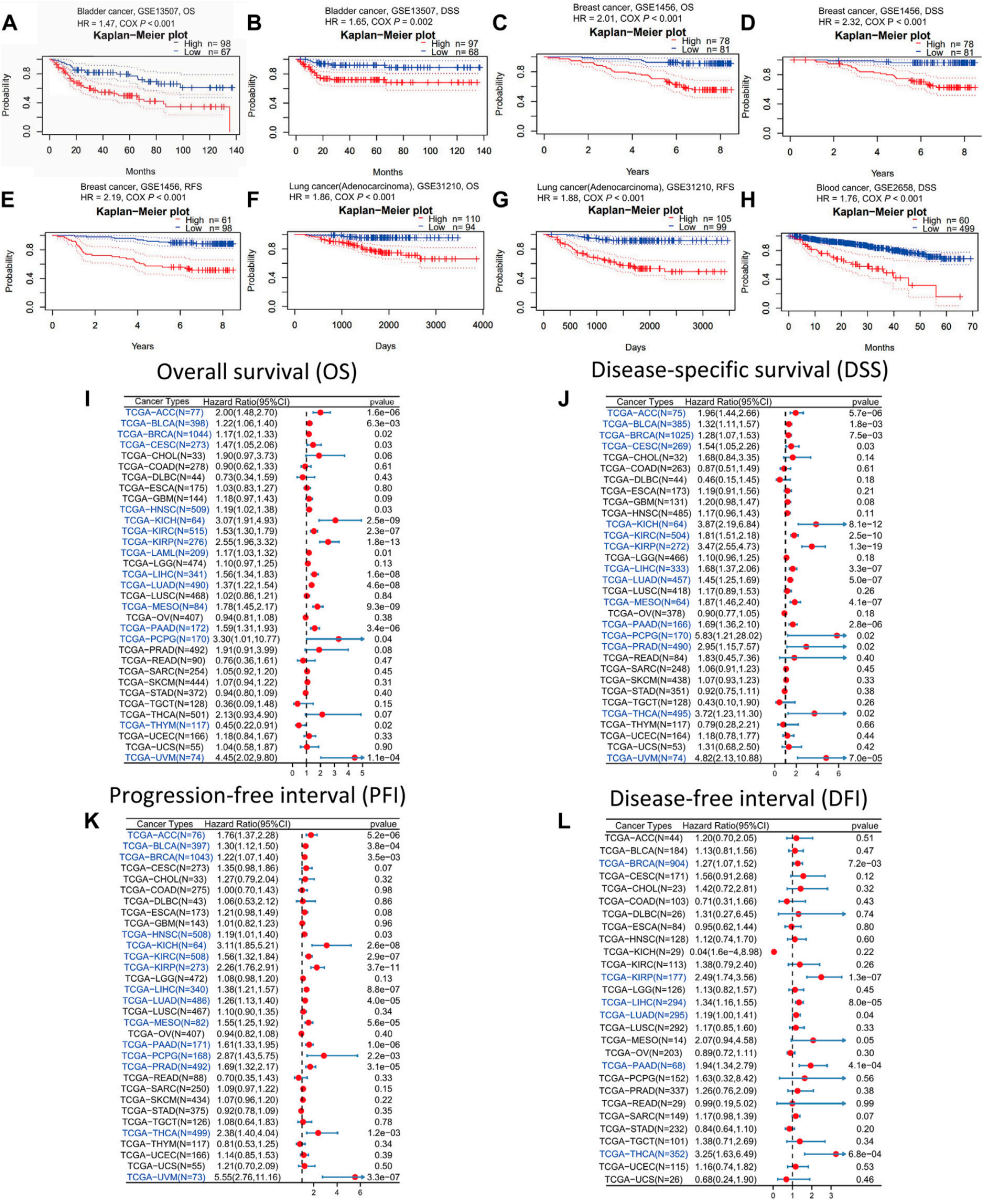
Survival analysis of ANLN across different cancer types in the GEO and TCGA datasets. Kaplan-Meier plots of ANLN in eight cohorts including GSE13507, OS **(A)**; GSE13507, DSS **(B)**; GSE1456, OS **(C)**; GSE1456, DSS **(D)**; GSE1456, RFS **(E)**; GSE31210, OS **(F)**; GSE31210, RFS **(G)**; GSE2658, DSS **(H)**. Forest plots demonstrating the relationship between ANLN expression and patient OS **(I)**, DSS **(J)**, PFI **(K)**, and DFI **(L)**. Statistically significant results are marked in blue.

As stated above, in the vast majority of tumor types, ANLN expression was dramatically increased. To understand whether ANLN expression affected the prognosis of tumor patients, we utilized the PrognoScan database to get an ANLN expression-based survival analysis of cancer patients. After analyzing the eighteen independent prognostic cohorts derived from fourteen datasets (GSE13507, GSE1456, GSE31210, GSE2658, GSE19234, GSE4412, GSE1379, GSE3494, GSE9195, GSE9893, GSE12276, GSE3141, GSE8894, and GSE31213), we discovered that higher ANLN expression was linked to worse prognosis (Cox *p* < 0.05; Figures 3A–H, Supplementary Figure S4A). Additionally, we included 20 different datasets from GEO, and as illustrated in Supplementary Figures S4B, C, we found that ANLN expression was negatively correlated with patient prognosis.

Then we downloaded the TCGA RNA-seq data and accompanied clinical information from UCSC Xena to have a deeper understanding of the prognostic value of ANLN. Using Cox proportional hazards model, we looked into the ANLN-related survival (OS, DSS, PFI, and DFI). In OS analysis, we observed that high ANLN expression was a detrimental prognostic factor in ACC, BLCA, BRCA, CESC, HNSC, KICH, KIRC, KIRP, LAML, LIHC, LUAD, mesothelioma (MESO), PAAD, PCPG, THYM, and uveal melanoma (UVM) (Figure 3I). Regarding DSS of pan-cancer, the ANLN played a risk role for patients with ACC, BLCA, BRCA, CESC, KICH, KIRC, KIRP, LIHC, LUAD, MESO, PAAD, PCPG, PRAD, THCA, and UVM (Figure 3J). For PFI analysis, high ANLN expression was associated with short PFI in ACC, BLCA, BRCA, HNSC, KICH, KIRC, KIRP, LIHC, LUAD, MESO, PAAD, PCPG, PRAD, THCA, and UVM (Figure 3K). Regarding the association between ANLN and DFI, we found that upregulation of ANLN was related to poorer DFI prognosis in BRCA, KIRP, LIHC, LUAD, PAAD, and THCA (Figure 3L).

We got ANLN-related survival (OS and RFS) through the Kaplan-Meier plotter database to further verify our results. Higher ANLN expression heralded shorter OS and RFS in BRCA, CESC, KIRP, LIHC, LUAD, PAAD, SARC, THCA, and UCEC. On the contrary, in esophageal squamous cell carcinoma (ESCC) and OV, patients with ANLN high expression had significant and favourable survival outcomes (Supplementary Figures S5, S6). Based on the above results, we could deduce that ANLN could be utilized as a prognostic biomarker in most cancer types.

### ANLN has predictive value in pan-cancer

**FIGURE 4:**
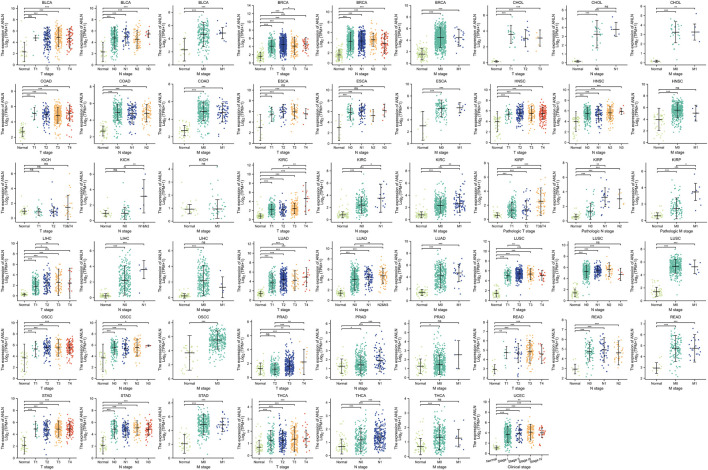
Association between ANLN expression and tumor stages (T, N, M, and clinical stages). *p < 0.05, **p < 0.01, ***p < 0.001. Ns, not statistically significant.

According to our study on the tumor stage relevance, there were 17 types of cancer with a significant increase in ANLN expression in early tumor stages, including BLCA, BRCA, CHOL, COAD, ESCA, HNSC, KIRC, KIRP, LIHC, LUAD, LUSC, oral squamous cell carcinoma (OSCC), PRAD, READ, STAD, THCA, and UCEC (Figure 4). This suggested that ANLN might serve as an important clinical marker for early cancer detection.

**FIGURE 5:**
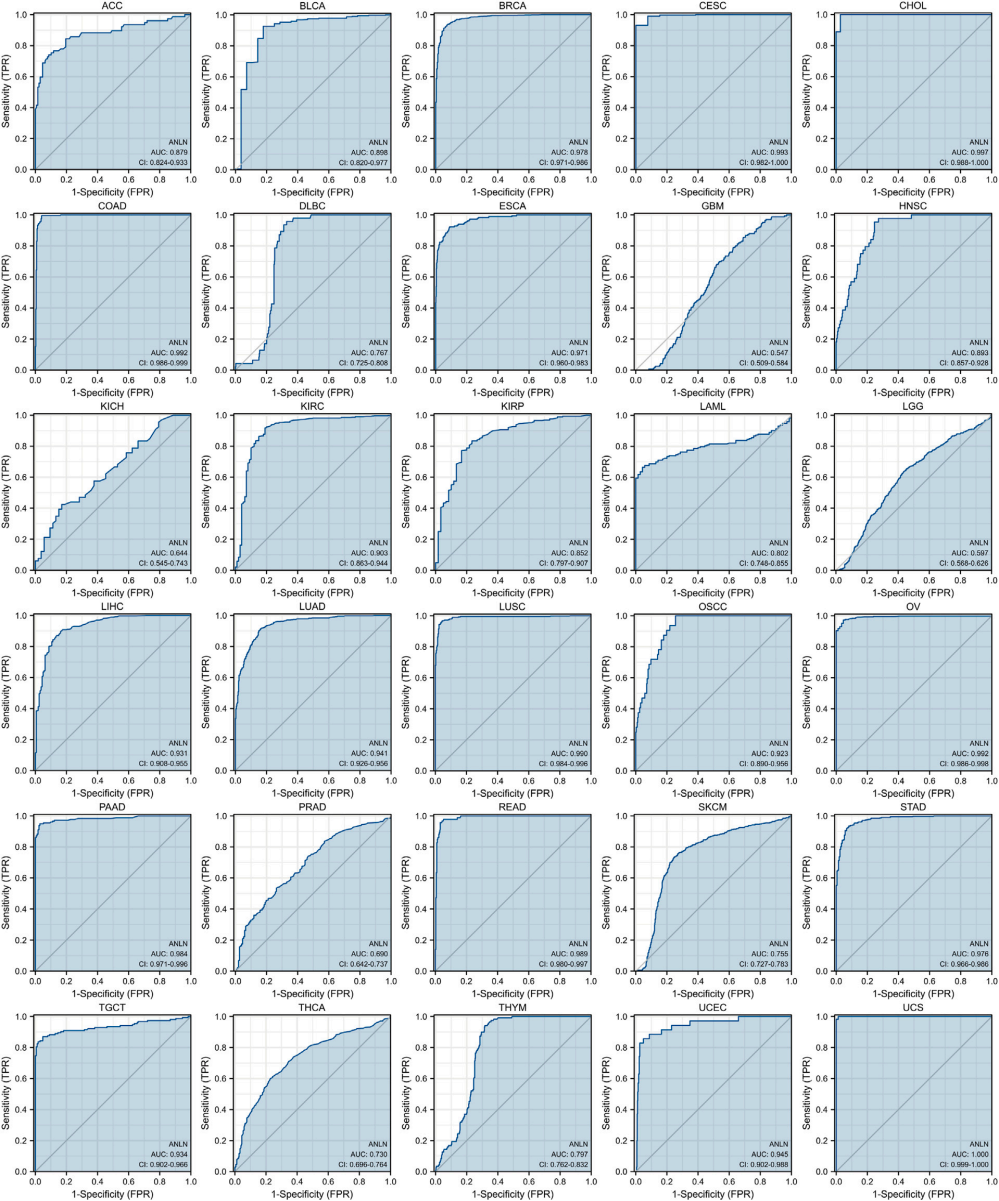
ROC curves indicate that ANLN has good discrimination power between tumor and normal tissues in pan-cancer. The *X*-axis represents the false positive rate (FPR), and the *Y*-axis represents the true positive rate (TPR). The larger the area under the curve (AUC), the higher the predictive accuracy.

The ROC curve was then introduced and we revealed the predictive value of ANLN in pan-cancer. As could be seen in Figure 5, ANLN had a certain accuracy (AUC = 0.7–0.9) in predicting 9 cancer types, including ACC (AUC = 0.879), BLCA (AUC = 0.898), DLBC (AUC = 0.767), HNSC (AUC = 0.893), KIRP (AUC = 0.852), LAML (AUC = 0.802), SKCM (AUC = 0.755), THCA (AUC = 0.730), and THYM (AUC = 0.797). Furthermore, ANLN showed a high accuracy in predicting BRCA (AUC = 0.978), CESC (AUC = 0.993), CHOL (AUC = 0.997), COAD (AUC = 0.992), ESCA (AUC = 0.971), KIRC (AUC = 0.903), LIHC (AUC = 0.931), LUAD (AUC = 0.941), LUSC (AUC = 0.990), OSCC (AUC = 0.923), OV (AUC = 0.992), PAAD (AUC = 0.984), READ (AUC = 0.989), STAD (AUC = 0.976), TGCT (AUC = 0.934), UCEC (AUC = 0.945), and UCS (AUC = 1.000). Yet, the predictive accuracy of ANLN was low in predicting GBM (AUC = 0.547), KICH (AUC = 0.644), LGG (AUC = 0.597), and PRAD (AUC = 0.690).

Overall, ANLN had moderate to strong power to predict tumor tissues and normal tissues except for a small number of cancer types like GBM, KICH, LGG, and PRAD.

### Functional enrichment analyses reveal that ANLN is involved in DNA-replication-related processes

**FIGURE 6:**
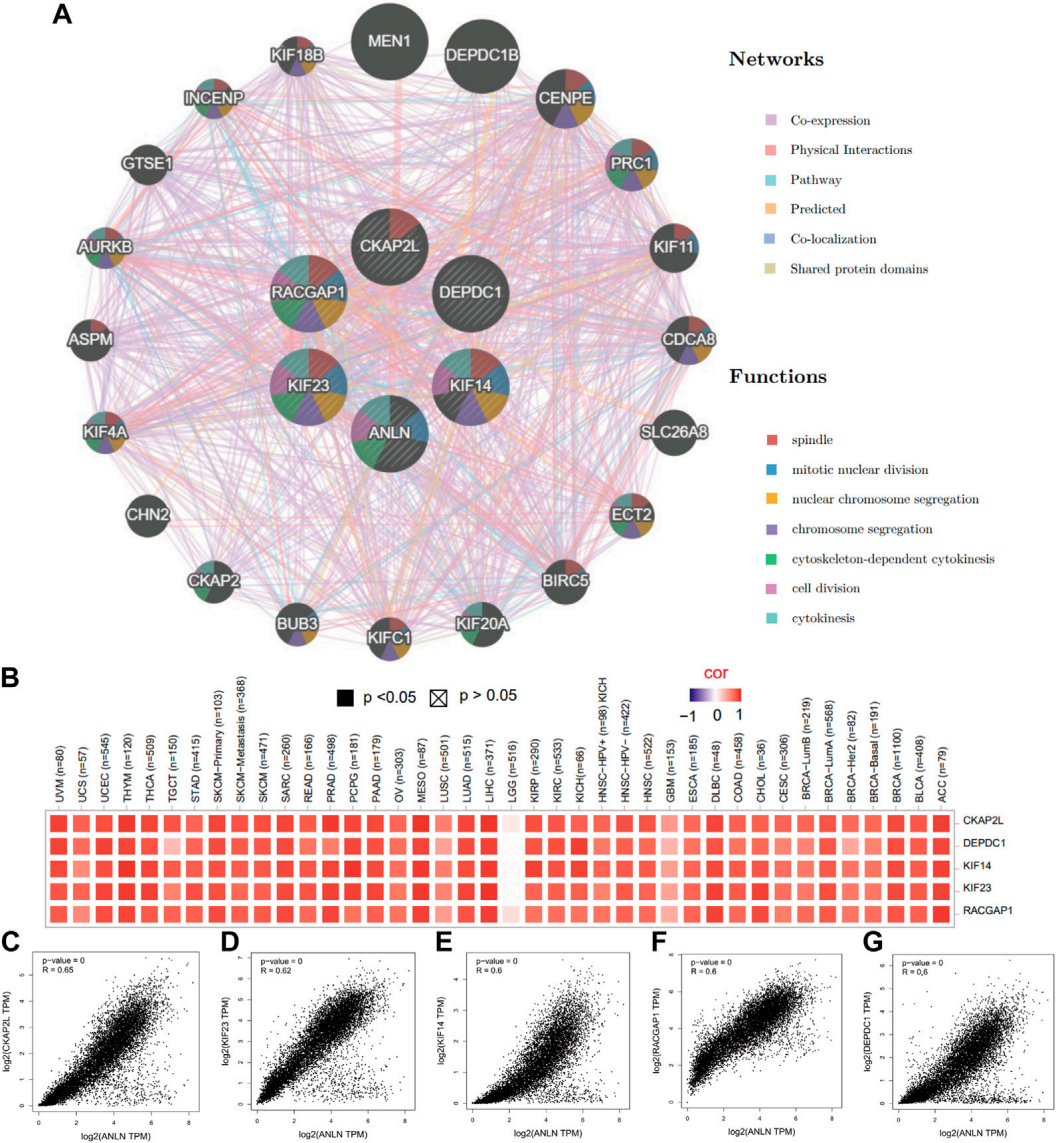
The top five ANLN co-expressed genes in pan-cancer. **(A)** A protein-protein interaction network of ANLN and co-expressed genes using GeneMANIA. Different color represents different networks and functions. **(B)** Heatmap of relations between ANLN and the top five genes in diverse TCGA tumors. Scatterplots showing the correlation between ANLN and CKAP2L **(C)**, KIF23 **(D)**, KIF14 **(E)**, RACGAP1 **(F)**, and DEPDC1 **(G)** in pan-cancer.

To gain a thorough knowledge of ANLN’s possible molecular processes in tumor development and progression, we explore the enrichment analysis of ANLN co-expressed genes. First, we obtained the top 100 ANLN co-expressed genes after combing all TCGA tumor expression data. The top five genes were CKAP2L (cytoskeleton-associated protein 2-like) (Figure 6C, R = 0.65, *p*-value = 0), KIF23 (kinesin family member 23) (Figure 6D, R = 0.62, *p*-value = 0), KIF14 (kinesin family member 14) (Figure 6E, R = 0.60, *p*-value = 0), RACGAP1 (Rac GTPase activating protein 1) (Figure 6F, R = 0.60, *p*-value = 0), and DEPDC1 (DEP domain containing 1) (Figure 6G, R = 0.60, *p*-value = 0). Their expression correlations with ANLN in pan-cancer were visualized in Figure 6B as a heatmap.

Next, we performed an interaction network in GeneMANIA to find potential genes which shared functional similarities with ANLN, CKAP2L, KIF23, KIF14, RACGAP1, and DEPDC1. We obtained 20 similar genes, and their functions were predominant DNA replication-related. The top seven functions with the lowest false discovery rate (FDR) included spindle, mitotic nuclear division, nuclear chromosome segregation, chromosome segregation, cytoskeleton-dependent cytokinesis, cell division, and cytokinesis (Figure 6A).

**FIGURE 7:**
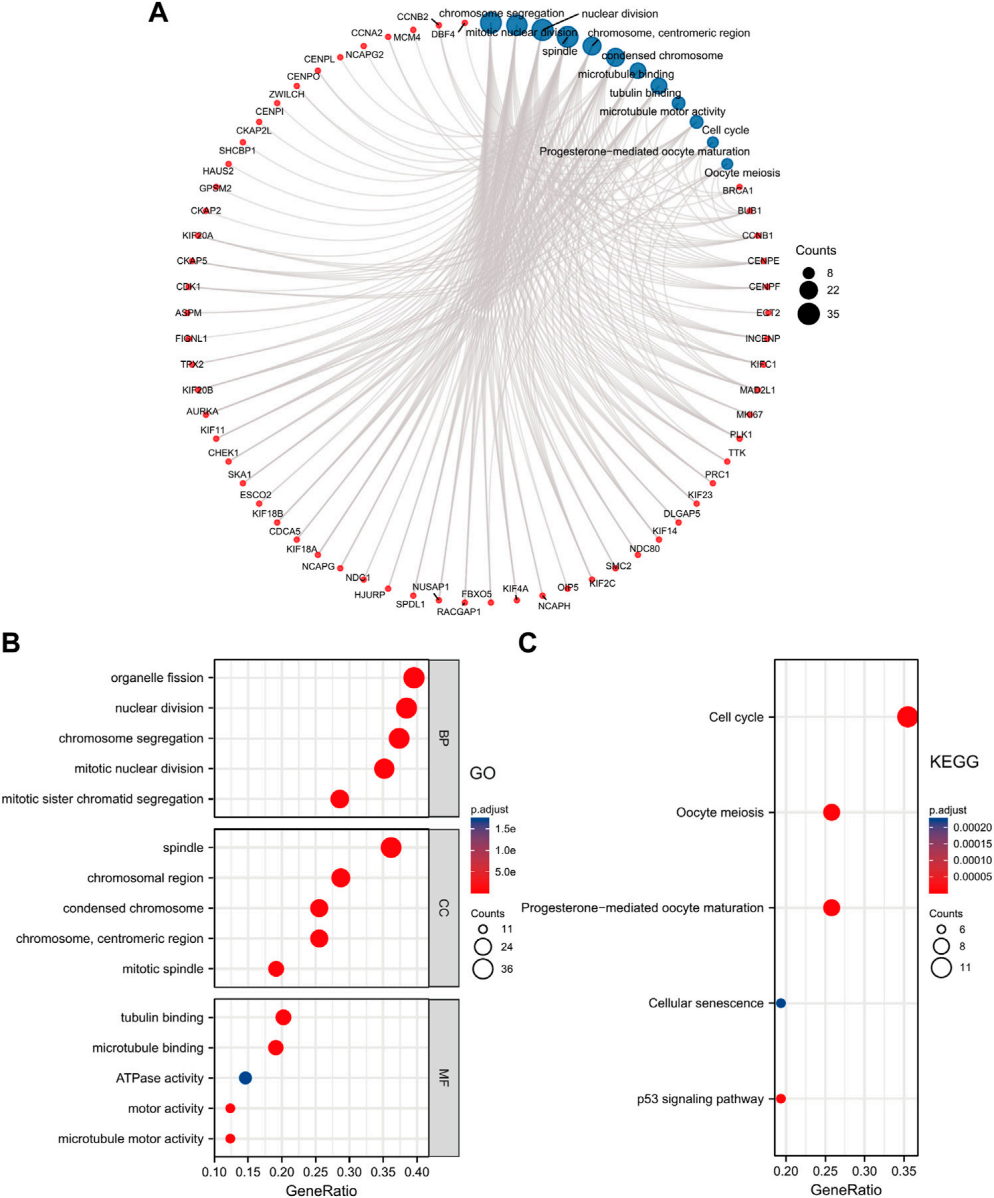
GO and KEGG enrichment analysis of ANLN co-expressed genes. **(A)** Visual network of GO and KEGG analyses. **(B)** GO analysis shows the top five enriched terms of BP, CC, and MF. **(C)** KEGG analysis shows the top five enriched pathways. The color and size of the circle represent the adjusted *p*-value and counts number, respectively.

Additionally, we performed GO and KEGG Pathways analyses based on the ANLN co-expressed top 100 genes. The results revealed that the BP was primarily involved in organelle fission, nuclear division, chromosome segregation, mitotic nuclear division, and mitotic sister chromatid segregation. The CC was mainly enriched in the spindle, chromosomal and centromeric region, condensed chromosome, and the mitotic spindle. The MF contained tubulin binding, microtubule-binding, ATPase activity, motor activity, and microtubule motor activity (Figure 7B). The KEGG pathways analysis elucidated that these genes highly likely participate in the cell cycle processes, oocyte meiosis, progesterone-mediated oocyte maturation, cellular senescence, and p53 signaling pathway (Figure 7C). We conducted a visual network of GO and KEGG analyses to improve visualization, as shown in Figure 7A.

**FIGURE 8:**
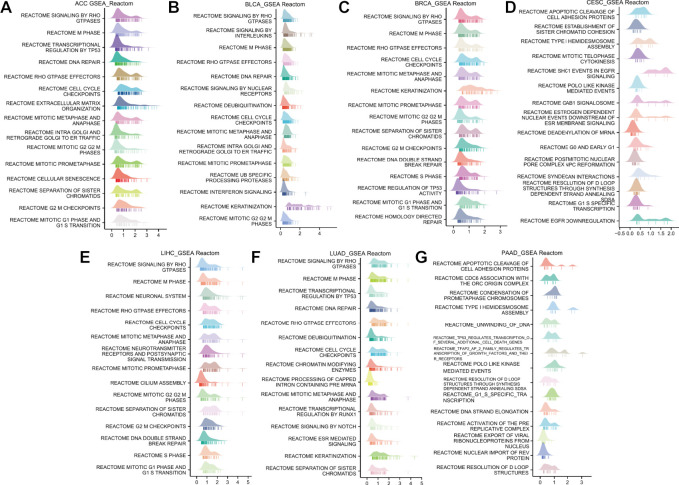
GSEA functional enrichment analysis of ANLN in 7 cancers. The top 15 Reactom pathways of ANLN in ACC **(A)**, BLCA **(B)**, BRCA **(C)**, CESC **(D)**, LIHC **(E)**, LUAD **(F)**, and PAAD **(G)**. LogFC values are distributed according to the number of core molecules in each gene set, and the *Y*-axis represents each gene set.

To explore the potential pathways of ANLN participating in pan-cancer, we then conducted a GSEA analysis based on the Reactome pathway database. A total of 7 cancer types whose prognoses were inversely correlated with ANLN expression were incorporated in our analysis, including ACC, BLCA, BRCA, CESC, LIHC, LUAD, and PAAD. As depicted in Figure 8, our GSEA results demonstrated that ANLN was likely to be actively involved in cell cycle-related and DNA replication-related processes, like the M phase, cell cycle checkpoints, mitotic prometaphase, and mitotic G2/M phases. TP53 and Rho GTPase signaling was also associated with ANLN expression (Figures 8A–G).

Finally, we studied the differential ANLN expression level in pan-cancer between wild-type (WT) TP53 and mutated TP53 groups. It was not difficult to find that in most cancer types, ANLN expression was significantly higher in patients with mutated TP53. While for patients with DLBC or LGG, the WT TP53 group tended to have lower ANLN expression (Supplementary Figures S7A, B). In short, it was reasonable to infer that ANLN exerted its oncogenic effects by affecting DNA replication-related pathways and regulating the activity and stability of TP53.

### ANLN correlates with immune infiltration and immune response in pan-cancer

**FIGURE 9:**
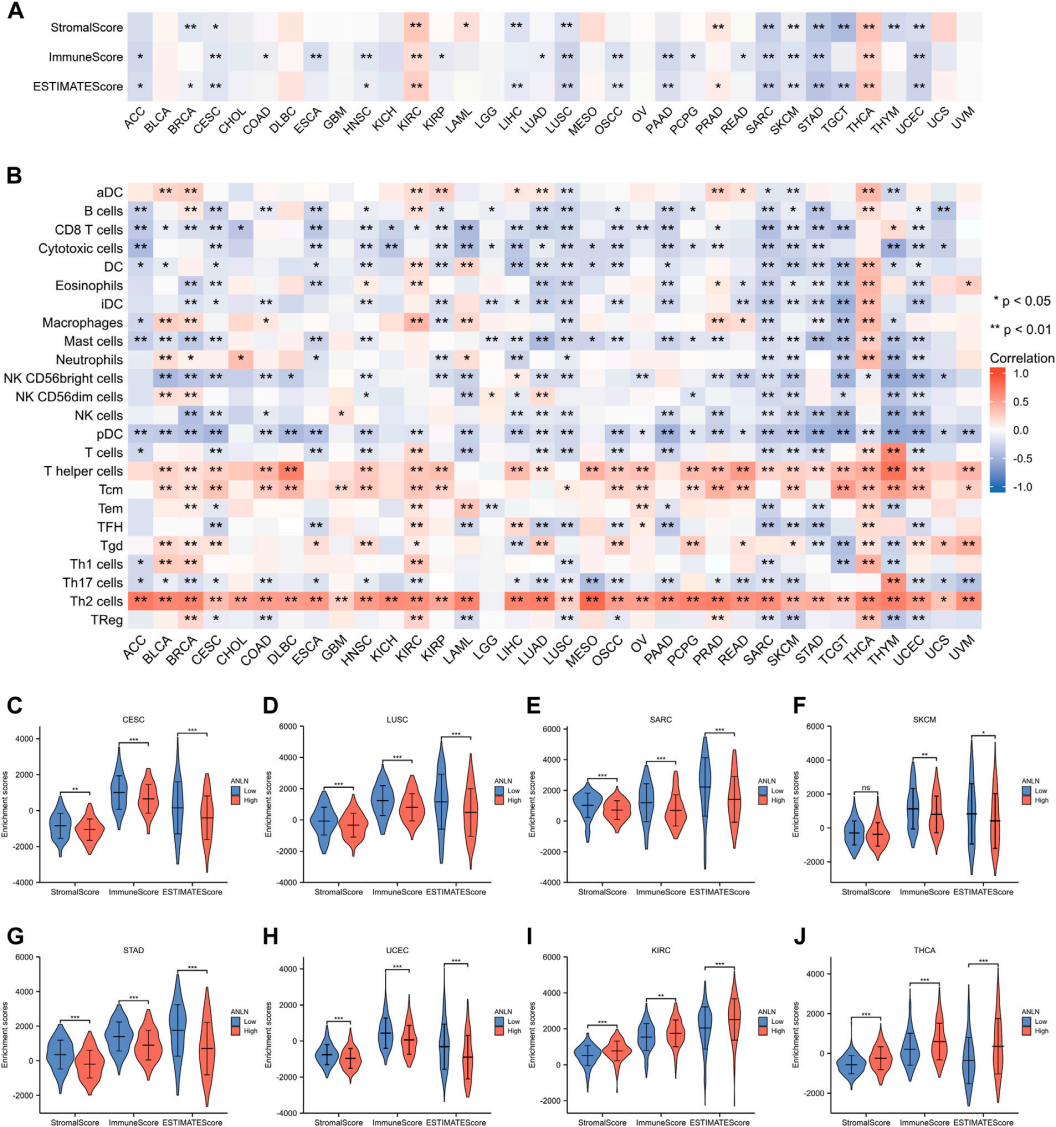
Associations between immune cell infiltration levels and ANLN expression in pan-cancer. **(A)** The correlation of ANLN expression and immune infiltration using the ESTIMATE algorithm. **(B)** The correlation of ANLN expression and immune infiltration using the ssGSEA algorithm. The distribution of immune scores, stromal scores, and ESTIMATE scores between ANLN low and high groups in CESC **(C)**, LUSC **(D)**, SARC **(E)**, SKCM **(F)**, STAD **(G)**, UCEC **(H)**, KIRC **(I)**, and THCA **(J)**.

The ssGSEA and ESTIMATE methods were used to assess the relationships between ANLN expression and immune infiltration. In most cancer types, ANLN expression was found to be substantially linked with immune cell infiltration levels (Figures 9A,B). Specifically, ANLN expression was negatively correlated with the stroma score, immune score, and ESTIMATE scores in six cancers, including CESC, LUSC, SARC, SKCM, STAD, and UCEC. While in KIRC and THCA, positive and significant correlations were observed between ANLN and these three indexes (Figure 9A). After dividing patients according to median ANLN expression, we observed that ANLN high expression presented lower stromal, immune, and ESTIMATE scores in CESC (Figure 9C), LUSC (Figure 9D), SARC (Figure 9E), STAD (Figure 9G), and UCEC (Figure 9H)). In SKCM, the immune and ESTIMATE scores were also lower in ANLN high group, while the stromal score showed no difference (Figure 9F). For KIRC and THCA, patients with high ANLN expression possessed higher stromal, immune, and ESTIMATE scores (Figures 9I,J). In addition, the heatmap in Figure 9B illustrated a significant correlation between the infiltration of T helper cells, central memory T cells (Tcm), and Th2 cells and ANLN expression. In contrast, the infiltration of other immune cells in most cancers was negatively correlated with ANLN expression except for KIRC and THCA, which was generally consistent with the results in Figure 9A.

**FIGURE 10:**
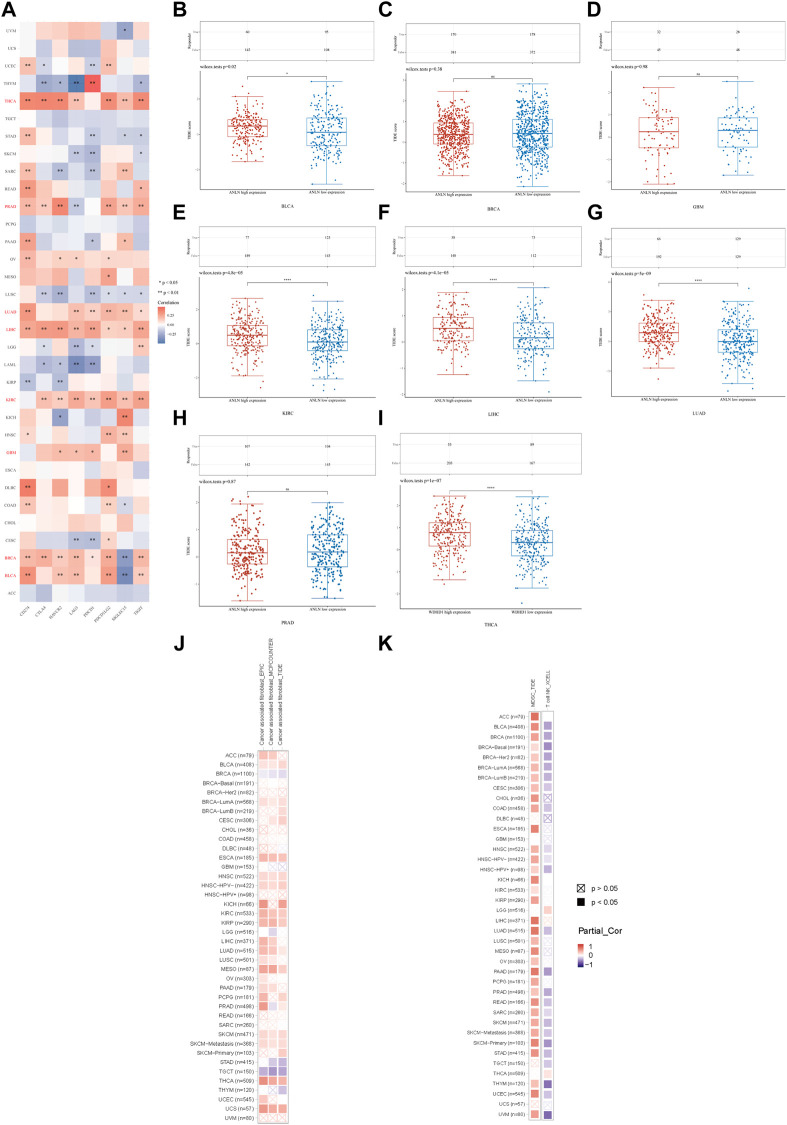
**(A)** Correlation between ANLN and immunoinhibitors in pan-cancer. TIDE score of ANLN high and low expression groups in BLCA **(B)**, BRCA **(C)**, GBM **(D)**, KIRC **(E)**, LIHC **(F)**, LUAD **(G)**, PRAD **(H)**, and THCA **(I)**. **(J)** Correlation of the CAFs infiltration level and ANLN expression in cancers. **(K)** Correlation of the MDSC (left), T cell NK (right) infiltration level and ANLN expression in cancers (*p < 0.05, **p < 0.01, ***p < 0.001).

Moreover, we evaluated the association between ANLN and immunoinhibitors. On the whole, ANLN was correlated with the expression of immunoinhibitors in pan-cancer. For patients with BLCA, BRCA, GBM, KIRC, LIHC, LUAD, PRAD, and THCA, statistically significant and positive correlations could be observed between ANLN expression and most immunoinhibitors (Figure 10A). To better understand the ANLN expression effect on ICB treatment, we acquired the TIDE scores of ANLN in the eight cancers mentioned above. We found that the ANLN high expression group possessed higher TIDE scores in BLCA (Figure 10B), KIRC (Figure 10E), LIHC (Figure 10F), LUAD (Figure 10G), and THCA (Figure 10I), which suggested that ANLN might impair ICB response by promoting immune escape in these tumors. No significant difference was observed in BRCA (Figure 10C), GBM (Figure 10D), and PRAD (Figure 10H).

In the last, we used TIMER2.0 for further evaluation. We discovered the infiltration of CAFs (cancer-associated fibroblasts) in BLCA, ESCA, HNSC, KIRC, KIRP, LUAD, MESO, SKCM, THCA, and UCS positively correlated with ANLN expression. However, the relationship between ANLN and CAFs in BRCA and TGCT was negative (Figure 10J). The infiltration levels of MDSC (myeloid-derived suppressor cells) and nature kill T cells (T cell NK) positively and negatively correlated with ANLN expression in most cancer types, respectively (Figure 10K). To conclude, ANLN had an essential role in immune infiltration and ICB treatment response.

### ANLN expression differs in different immune and molecular subtypes

**FIGURE 11:**
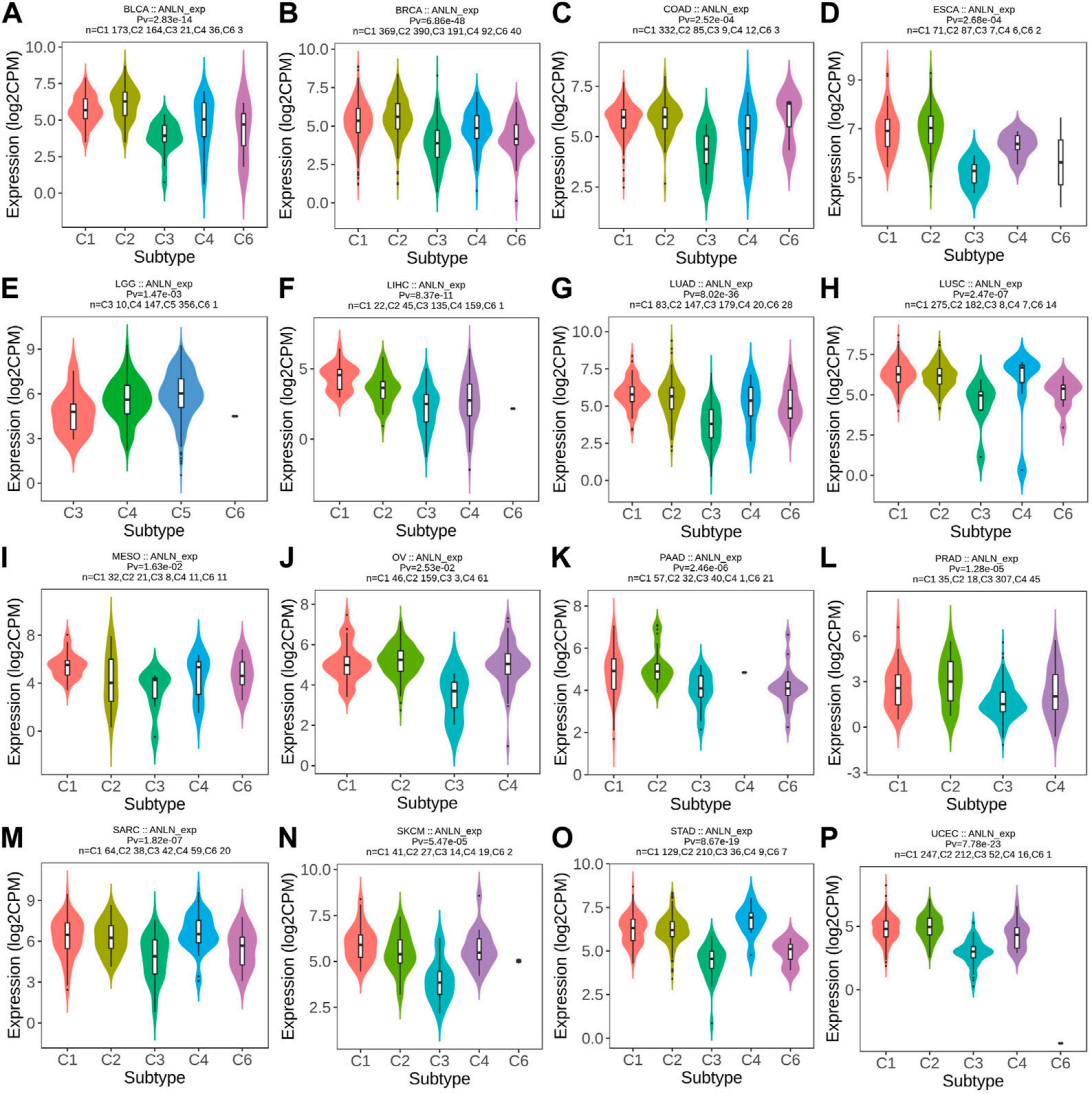
Correlations between immune subtypes and ANLN expression across TCGA tumors. **(A)** BLCA; **(B)** BRCA; **(C)** COAD; **(D)** ESCA; **(E)** LGG; **(F)** LIHC; **(G)** LUAD; **(H)** LUSC; **(I)** MESO; **(J)** OV; **(K)** PAAD; **(L)** PRAD; **(M)** SARC; **(N)** SKCM; **(O)** STAD; **(P)** UCEC. C1 (wound healing), C2 (IFN-g dominant), C3 (inflammatory), C4 (lymphocyte deplete), C5 (immunologically quiet), and C6 (TGF-b dominant).

By TISIDB, we explored the differential ANLN expression in different immune and molecular subtypes in pan-cancer. As depicted in Figure 11A–P and Supplementary Figures S8A–G, ANLN expression was significantly associated with different immune subtypes of 23 cancers and in BLCA, BRCA, ESCA, LIHC, LUAD, LUSC, MESO, OV, PAAD, PRAD, SARC, SKCM, STAD, UCEC, HNSC, KIRC, and READ, ANLN expression tended to be relatively higher in C1 (wound healing) and C2 (INF-gamma dominant) immune subtypes. While in almost all cancer types, ANLN expression was less expressed in the C3 (inflammatory) immune subtype, a subtype featured with the best patient’ survival outcomes, as previous work has demonstrated (Thorsson et al., 2018).

**FIGURE 12:**
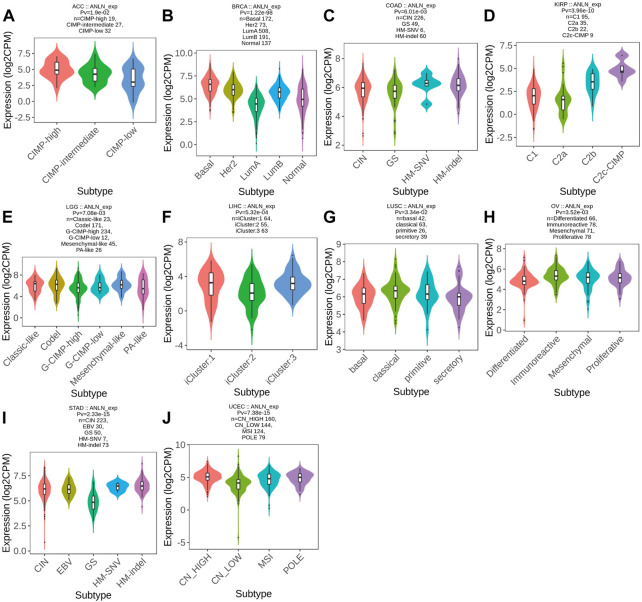
Correlations between molecular subtypes and ANLN expression across TCGA tumors. **(A)** ACC; **(B)** BRCA; **(C)** COAD; **(D)** KIRP; **(E)** LGG; **(F)** LIHC; **(G)** LUSC; **(H)** OV; **(I)** STAD; **(J)** UCEC.

Meanwhile, ANLN was differentially expressed in different molecular subtypes of ten cancer types. ANLN expression was highest in the molecular subtype of CIMP-high in ACC (Figure 12A), Basal for BRCA (Figure 12B), HM-SNV for COAD (Figure 12C), C2c-CIMP for KIRP (Figure 12D), Codel and Mesenchymal-like in LGG (Figure 12E), iCluster:1 and 3 for LIHC (Figure 12F), classical for LUSC (Figure 12G), immunoreactive for OV (Figure 12H), HM-SNV and HM-indel for STAD (Figure 12I), and CN_High in UCEC (Figure 12J). To conclude, the expression of ANLN varied by immune and molecular subtypes.

### ANLN is an independent prognostic factor in certain cancers

To determine risk factors that influence patients’ OS, we then conducted univariate and multivariate regression analyses in seven cancer types, the OS of which was previously demonstrated to be associated with ANLN expression, including ACC, BLCA, BRCA, CESC, LIHC, LUAD, and PAAD. For ACC, multivariate analysis indicated that T stage (T3/T4, hazard ratio (HR) = 4.99, *p*-value = 0.004), new event (with new event, HR = 5.42, *p*-value = 0.008), and ANLN expression (high ANLN, HR = 2.83, *p*-value = 0.037) could serve as independent prognostic factors that associated with patients’ OS (Supplementary Table S2A). For BLCA, primary therapy outcome (partial response (PR)/complete response (CR), HR = 0.42, *p*-value = 0.003) and ANLN expression (high ANLN, HR = 1.87, *p*-value = 0.022) were independent prognostic factors (Supplementary Table S2B). For BRCA, N stage (N1, HR = 1.60, *p*-value = 0.043), age (>60, HR = 2.20, *p*-value < 0.001), ANLN expression (high ANLN, HR = 1.58, *p*-value = 0.015) were independent prognostic factors (Supplementary Table S2C). For LIHC, T stage (T3/T4, HR = 2.28, *p*-value < 0.001), tumor status (with tumor, HR = 1.91, *p*-value = 0.007), ANLN expression (high ANLN, HR = 1.61, *p*-value = 0.042) were independent prognostic factors (Supplementary Table S2E). For LUAD, primary therapy outcome (PR/CR, HR = 0.324, *p*-value < 0.001), ANLN expression (high ANLN, HR = 2.023, *p*-value < 0.001) were independent prognostic factors (Supplementary Table S2F). For PAAD, N stage (N1, HR = 2.00, *p*-value = 0.021), ANLN expression (high ANLN, HR = 1.77, *p*-value = 0.014) were independent prognostic factors (Supplementary Table S2G). However, in CESC, risk factors with a *p*-value of less than 0.1 included primary therapy outcome (PR/CR, HR = 0.26, *p*-value = 0.097), and ANLN expression (high ANLN, HR = 2.92, *p*-value = 0.085) (Supplementary Table S2D). No risk factors were independently correlated with the OS of patients with CESC.

**FIGURE 13:**
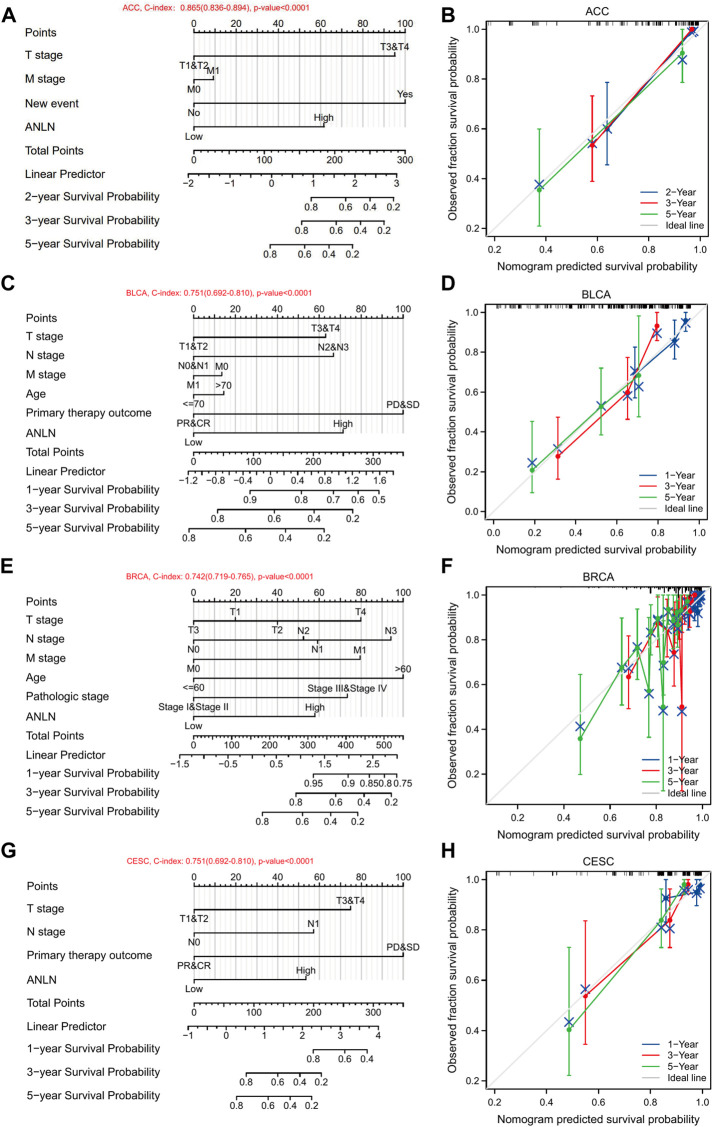
Nomograms and calibration curves predicting patient OS in 7 cancers. Nomograms of ACC **(A)**; BLCA **(C)**; BRCA **(E)**; CESC **(G)**. Calibration curves of ACC **(B)**; BLCA **(D)**; BRCA **(F)**; CESC **(H)**. The horizontal and vertical coordinates are the model predicted and actually observed survival probability, respectively. The closer each line is to the ideal line, the better the model.

Then we conducted nomograms and calibrations using the variables with *p*-values < 0.1 in the univariate analysis of the seven cancer types. In ACC, the C-index of the nomogram was 0.865 (0.836–0.894) (Figure 13A). In BLCA, the C-index of the nomogram was 0.751 (0.692–0.810) (Figure 13C). In BRCA, the C-index of the nomogram was 0.742 (0.719–0.765) (Figure 13E). In CESC, the C-index was 0.751 (0.692–0.810) (Figure 13G). In LIHC, the C-index was 0.659 (0.622–0.695) (Supplementary Figure S9A). In LUAD, the C-index was 0.727 (0.700–0.754) (Supplementary Figure S9C). In PAAD, the C-index was 0.661 (0.628–0.695) (Supplementary Figure S9E). The corresponding calibrations of each nomogram were performed to evaluate the model’s accuracy. Overall, the calibration curves were close to the ideal line, which Figures 13B,D,F,H signified a good fit between predicted and observed OS in the seven cancer types (Figures 13B,D,F,H, Supplementary Figures S9B,D,F). Accordingly, ANLN could be used to predict patient prognosis independently in some tumors.

### ANLN correlates with tumor heterogeneity and tumor stemness

**FIGURE 14:**
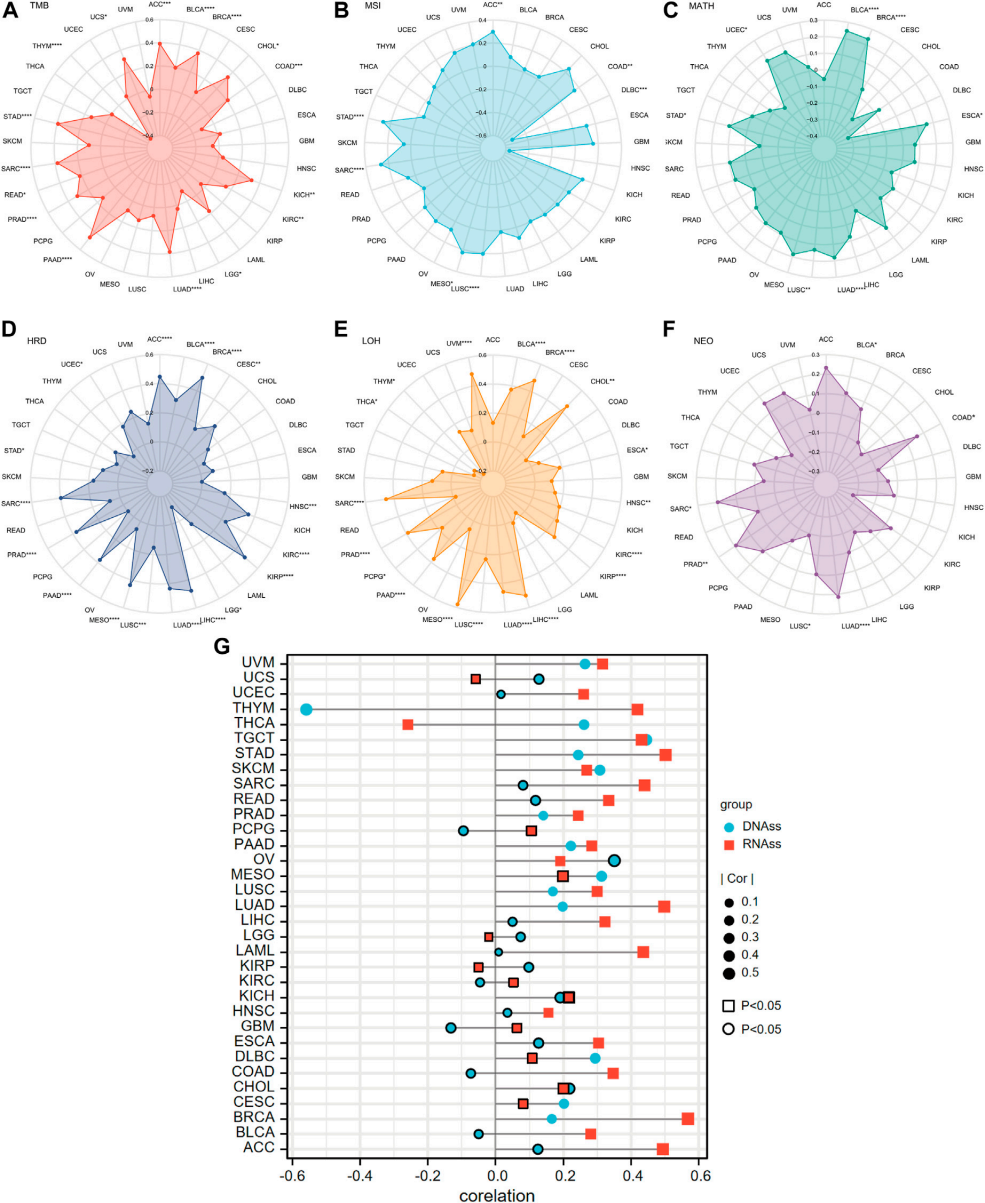
Correlations of ANLN expression and tumor heterogeneity including **(A)** TMB; **(B)** MSI; **(C)** MATH; **(D)** HRD; **(E)** LOH; **(F)** NEO in pan-cancer. **(G)** Correlation of tumor stemness and ANLN expression in pan-cancer. Different shapes and colors represent different tumor stemness indexes. The graph without peripherally bolded means a *p*-value of less than 0.05.

Overall, ANLN expression was positively correlated with the TMB of 14 cancer types, including ACC, BLCA, BRCA, CHOL, COAD, KICH, KIRC, LUAD, PAAD, PRAD, READ, SARC, STAD, and UCS, and negatively correlated with TMB in LGG and THYM (Figure 14A). In ACC, COAD, LUSC, MESO, SARC, and STAD, ANLN showed a significant and positive correlation with MSI, while in DLBC, the correlation was significant and negative (Figure 14B). Higher MATH was accompanied by high ANLN expression for patients with BLCA, BRCA, ESCA, LUAD, LUSC, STAD, and UCEC (Figure 14C). The expression trend of ANLN was consistent with HRD in ACC, BLCA, BRCA, CESC, HNSC, KIRC, KIRP, LIHC, LUAD, LUSC, MESO, PAAD, PRAD, SARC, STAD, and UCEC. The opposite trend between ANLN expression and HRD was observed in LGG (Figure 14D). In addition, Figure 14E depicted a significantly positive relationship between LOH and ANLN expression in BLCA, BRCA, CHOL, ESCA, HNSC, KIRC, KIRP, LIHC, LUAD, LUSC, MESO, PAAD, PCPG, PRAD, SARC, and UVM. However, in THCA and THYM, the relationship was statistically formulated to be negative (Figure 14E). At last, six cancer types showed a positive and significant correlation with NEO: BLCA, COAD, LUAD, LUSC, PRAD, and SARC (Figure 14F).

Two types of indexes that quantified tumor stemness were introduced in our investigation. They were DNAss and RNAss. The association between ANLN expression and tumor stemness was subsequently performed. What could be concluded from Figure 14G was that in most cases, ANLN expression had positive relation with at least one stemness index in all cancer types. Accordingly, ANLN may affect the stemness and heterogeneity of tumor cells, thus playing a carcinogenic role.

## Discussion

As described above, anillin protein encoded by the ANLN gene was a highly conserved protein with a muti-structural domain. Anillin is mainly localized in the skeleton and nucleus and it is indispensable for cell division through recruiting and binding to essential proteins in mitosis, including F-actin, Myosin II, and septin cytoskeleton. Due to its crucial roles in cell growth, migration, and cytoplasmic division, researchers have studied the role of ANLN in malignant tumors.

The current work started with a pan-cancer expression and survival investigation of ANLN, and the findings revealed that ANLN was upregulated in the majority of cancers. Additionally, we found that high ANLN expression was associated with poor survival in most cancers. Meanwhile, ANLN was upregulated in the early stages in 17 types of cancer and exhibited good predictive accuracy in many cancer types. As previous research has demonstrated, ANLN is ubiquitously overexpressed in diverse tumor tissues, except for brain tumors. ANLN expression increases as the tissues transition from normal to benign to malignant and, eventually, to metastatic disease (Hall et al., 2005). Recently, there has been sparse but accumulating evidence underpinning the association between ANLN expression and the development of different cancers. In addition to the cancers mentioned in the introduction section, functional experiments confirmed that the proliferation, migration, and invasion potential of BLCA cells was hindered by ANLN knockdown. The prognostic value of ANLN was validated by an additional cohort (Mannheim cohort) aside from TCGA-cohort (Zeng et al., 2017; Wu et al., 2019). In 2020, liver cancer is the third most common cancer worldwide (Sung et al., 2021). Current studies argued that ANLN downregulation incurred cell cycle arrest, thus inhibiting liver tumor cells proliferation assessed by both *in vitro* and in silicon analysis (Zhou et al., 2019; Zhang et al., 2021). Surprisingly, depriving of ANLN in cancer cell cytokinesis inhibited the development of liver tumors in mice without interfering with the regeneration of normal liver cells, which may provide superior referential value for future tumor treatment (Zhang et al., 2018). Apart from these cancers mentioned above, the carcinogenic effect of ANLN on cervical cancer, colorectal cancer, oral cancer, head and neck carcinoma, gastric cancer, and blood cancer was detailedly illustrated by functional experiments (Suzuki et al., 2005; Wang et al., 2016; Xu et al., 2019; Guo et al., 2021; Jia et al., 2021; Wang et al., 2021; Liu et al., 2022; Pan et al., 2022). Striking, our research has identified the prognostic value of ANLN in certain cancers (ACC, KICH, KIRC, KIRP, MESO, PCPG, PRAD, THCA, and UVM), which has hitherto not been reported by researchers through experiments. It is also of note that ANLN could be utilized as an independent prognostic factor in ACC, BLCA, BRCA, LIHC, LUAD, and PAAD, which has rarely been reported and greatly enriched the traditional predicting factors such as the TNM stage. To conclude, ANLN proved to be a promising marker for future cancer management.

Our GO and KEGG enrichment analysis using the ANLN co-expressed genes in pan-cancer revealed that ANLN participated in key biological processes involved in the cell cycle like organelle fission, nuclear division, and chromosome segregation. Besides this, as a critical component of organelle components in mitosis, ANLN functions as both a crucial binding protein to tubulin and a momentous regulator to the activity of ATPase and motor. Moreover, the functions of predicted proteins that might interact with ANLN were dominantly mitosis-relevant. It is now well accepted that the cell cycle is a meticulously regulated process in the human body, allowing for cell growth, genetic material replication, and cell division. Abnormal cell cycle machinery could be observed in virtually all tumor types and compromise a driving force of tumorigenesis (Suski et al., 2021). Our subsequent GSEA analysis also illustrated that ANLN was involved in the two critical events of the cell cycle, including replication of DNA and subsequent segregation between daughter cells. Previous studies in breast cancer cells have observed an increasing amount of cells stuck at the G2/M phase after ANLN knockdown (Zhou et al., 2015), and this is consistent with the observed function in regulating cell cycle phases of ANLN in our work. Additionally, ANLN might influence cycle checkpoints, which are indispensable for cells to avoid accumulating and amplifying genetic mistakes during cell division (Matthews et al., 2022). Besides affecting the cell cycle, recent studies have found previously underappreciated functions of nuclear ANLN, including controlling transcriptional programming and regulating the stemness and differentiation of cancer cells (Wang D. et al., 2020; Huang et al., 2021). To conclude, the functions of ANLN mentioned above could be a reasonable explanation for the enhanced cell proliferation in tumor cells.

In addition to boosting cell proliferation, ANLN is recognized as a potential cell migration stimulator, which has been proved by several *in vitro* experiments such as wound healing and Matrigel invasion assays. It is well documented that the accumulation of ANLN at the cell cortex regulates neuronal cells migration by stabilizing actin filaments (Tian et al., 2015). Besides, through binding to cytoskeletal regulators and regulating cell-cell junction, ANLN is likely to alter the cell-extracellular matrix (ECM) adhesions (Naydenov et al., 2021). These discoveries may serve as reasonable explanations for the pro-migratory effect of ANLN. It is also worth noting that our KEGG and GSEA analysis suggested a strong relationship between ANLN and the p53 signaling pathway. In more than 20 tumor types, especially in ACC, BLCA, LIHC, LUAD, PAAD, and UCEC (Supplementary Figure S7), patients with mutated TP53 tended to have higher ANLN expression levels compared to those with wild-type TP53. Numerous studies indicate that the most important tumor suppressor, p53, encoded by the TP53 gene, sustains normal cells growth and prevents tumor progression through its roles as a transcriptional factor and mitochondrial membrane permeabilization (Joerger and Fersht, 2016; Kastenhuber and Lowe, 2017). Traditionally, p53 is supposed to suppress tumorigenesis through involvement in cell cycle arrest, apoptosis, and DNA damage repair (Duffy et al., 2017; Engeland, 2018). TP53 is frequently mutated in most human malignancies, resulting in its tumor-suppressive function impairment. Usually, tumors with higher TP53 mutations progress more rapidly, respond poorly to anticancer therapy, and are linked with a dismal prognosis (Hu et al., 2021). Another significant pathway described in our KEGG and GSEA enrichment was cellular senescence. As a new perspective hallmark of cancer, cellular senescence is attracting more and more attention. Defined as a stable cell cycle arrest, cellular senescence occurs in diploid cells and hinders proliferative lifespan (Calcinotto et al., 2019). Cellular senescence plays a crucial role in different stages of human malignancies, including tumor formation, progression, and immune escape. It is characterized by the activation of senescence-associated secretory phenotype (SASP) (Calcinotto et al., 2019). Previous studies have long thought of cell senescence as a protection mechanism to fight against cancer cells. However, more and more evidence reveals that senescent cells contribute to tumour cells’ development and malignant biological behaviour (Faget et al., 2019). As far as we know, our research was the first to come up with the assumption that ANLN might affect cellular senescence in the malignant tumor, although the mechanism is still unclear and remains to be elucidated minutely.

Hardly any previous studies have addressed the critical relationship between ANLN and the tumor microenvironment (TME). Based on Spearman’s correlation of ANLN expression and the infiltration levels of immune cells, we found that ANLN was negatively correlated with immune infiltration in most cases. Especially compared with Th1 cells, ANLN showed stronger and positive correlations with Th2 cells. It seems that ANLN could skew the differentiation of Th1 cells towards the Th2 phenotype, which means shifting the immune response from antitumor to tumor-promoting (Ziani et al., 2018; Monteran and Erez, 2019). This seems to be a plausible explanation for the carcinogenesis of ANLN. Moreover, CAFs, MDSC, and NKT cell infiltration levels were strongly associated with ANLN expression, as shown in Figures 10J,K. CAFs are essential components of the TME and have been implicated in facilitating tumor cell progression by supporting growth, angiogenesis, drug resistance, and metastasis in most instances (Monteran and Erez, 2019; Joshi et al., 2021). Similarly, MDSC is a heterogeneous population of immature bone marrow cells. They inhibited the regular activity of T-cell and NK-cell and were described as the cornerstone of the immunosuppressive microenvironment that provided shelter for cancer from the patient’s immune system (Tesi, 2019; Law et al., 2020). These also support our hypothesis that ANLN could help tumors survive from human body immunological surveillance. Mechanistically, considering the fact that ANLN is closely linked to actomyosin cytoskeleton, which is required for the remodeling of ECM, and cell-cell adhesion, we, therefore, assume that, on the one hand, ANLN might alter the ECM component and limit the migration of immune cells in the TME, contributing to an immune-suppressive microenvironment that facilitates tumor cell survival, on the other hand, ANLN might mediate the contact between immunosuppressive cells (CAFs, MDSC) and immune effector cells (T and B-lymphocytes), thus exerting its immune suppressive and pro-tumorigenic functions (Calvo et al., 2013; Reyes et al., 2014; Law et al., 2020).

In the final, we observed that the expression of immunoinhibitors was closely related to ANLN in pan-cancer. In BLCA, BRCA, GBM, KIRC, LIHC, LUAD, PRAD, and THCA, most immunoinhibitors showed negative correlations with ANLN expression. Specifically, in BLCA, KIRC, LIHC, LUAD, and THCA, higher TIDE scores were observed in ANLN high expression group. Usually, it is considered that an increased tumor TIDE score is associated with a worse ICB response, as well as a lower likelihood of survival under anti-PD1 and anti-CTLA4 therapy (Jiang et al., 2018; Zhang et al., 2021). These findings suggested that ANLN might facilitate tumor immune invasion, and targeting ANLN could be a novel strategy for immunotherapy in these tumors. As we have already stated, cytokinesis is the primary biological function of ANLN. Recently, a substantial body of evidence has been arguing that aberrant cytokinesis contributes to tumor heterogeneity and genetic diversification, promoting tumor progression (Lens and Medema, 2019). These observations are consistent with our observations that ANLN has significant correlations with TMB, MSI, MATH, HRD, LOH, and NEO in pan-cancer. Cancer stem cells (CSCs) represent the cells that are given the potential for self-renewal and differentiation. They enhance metastatic tumor propensity and hinder the effectiveness of treatment (Saygin et al., 2019; Chen et al., 2021). In breast cancer, ANLN was reported to affect the stemness and differentiation of MCF10AneoT cells, and we also observed significant correlations between ANLN expression with DNAss, and RNAss in many tumors (Wang F. et al., 2020). These may be explained by the fact that ANLN is an actin-binding protein, and the nuclear actin regulates cells stemness and differentiation, as implicated in several types of research (Miyamoto et al., 2011; Sen et al., 2017). To conclude, abnormal ANLN expression may affect tumor cells’ stemness and genomic stability, thereby facilitating tumor progression.

Recently, research into pan-cancer is at a thrilling and crucial stage for exploring tumorigenesis and development. In our study, we provide an overview of ANLN’s roles in pan-cancer, which includes gene expression, prognostic value, molecular mechanisms, immunological roles, predictive value, and tumor heterogeneity, indicating that ANLN is a potential therapeutic biomarker for malignancies. There is no denying that our study has some limitations. Firstly, the data incorporated in our study mainly come from TCGA, GTEx, and GEO databases, which need further validation from other sources. Secondly, the detailed carcinogenesis mechanisms of ANLN in pan-cancer need to be fully addressed further by experiments conducted *in vitro* and *in vivo*. In general, our study contributes to uncovering the tumor-promoting effect of ANLN in diverse cancers, and we comprehensively describe the value of ANLN in the tumor microenvironment, patient prognosis and diagnosis.

## Data Availability

Publicly available datasets were analyzed in this study. These data can be found freely from TCGA data portal (https://portal.gdc.cancer.gov/) and GEO database (https://www.ncbi.nlm.nih.gov/geo/).

## Supplementary material

SUPPLEMENTARY FIGURE S1 ANLN mRNA expression between tumor and normal tissues in 36 independent cohorts from the GEO database. T is short for tumor tissues, and N is short for normal tissues (**p* < 0.05, ***p* < 0.01, ****p* < 0.001).

SUPPLEMENTARY FIGURE S2 ANLN promoter methylation level in pan-cancer with significance, using data from the UALCAN. **(A)** BLCA; **(B)** BRCA; **(C)** HNSC; **(D)** KIRP; **(E)** LIHC; **(F)** LUAD; **(G)** LUSC; **(H)** PRAD; **(I)** READ; **(G)** SARC; **(K)** STAD; **(L)** THYM; **(M)** UCEC; **(N)** THCA (**p* < 0.05, ***p* < 0.01, ****p* < 0.001).

SUPPLEMENTARY FIGURE S3 ANLN protein expression in pan-cancer with significance, using data from the UALCAN. **(A)** COAD; **(B)** GBM; **(C)** HNSC; **(D)** KIRC; **(E)** LIHC; **(F)** LUAD; **(G)** OV; **(H)** PAAD; **(I)** UCEC; **(J)** BRCA (**p* < 0.05, ****p* < 0.001).

SUPPLEMENTARY FIGURE S4 Survival analysis of ANLN in different GEO datasets. **(A)** 10 independent cohorts from the PrognoScan. **(B)** 20 independent cohorts from the GEO.

SUPPLEMENTARY FIGURE S5 Kaplan-Meier plots depict the associations between ANLN expression and patient OS in pan-cancer through the KM-plotter database.

SUPPLEMENTARY FIGURE S6 Kaplan-Meier plots depict the associations between ANLN expression and patient RFS in pan-cancer through the KM-plotter database.

SUPPLEMENTARY FIGURE S7 ANLN expression difference between WT TP53 and mutated TP53 groups in pan-cancer. **(A)** Heatmap shows the logFC and *p*-value. **(B)** ANLN expression of WT TP53 and mutated TP53 groups in ACC, BRCA, DLBC, KIRC, LIHC, LUAD, PAAD, and UCEC.

SUPPLEMENTARY FIGURE S8 Correlations between immune subtypes and ANLN expression across TCGA tumors. **(A)** ACC; **(B)** HNSC; **(C)** KICH; **(D)** KIRC; **(E)** READ; **(F)** TGCT; **(G)** THCA.

SUPPLEMENTARY FIGURE S9 Nomograms and calibration curves predicting patient OS in certain cancers. Nomograms of LIHC **(A)**; LUAD **(C)**; PAAD **(E)**. Calibration curves of LIHC **(B)**; LUAD **(D)**; PAAD **(F)**.

SUPPLEMENTARY TABLE S1 Detailed information of the GEO datasets incorporated in the study.

SUPPLEMENTARY TABLE S2 Univariate and multivariate Cox analysis of clinical parameters in ACC (A), BLCA (B), BRCA (C), CESC (D), LIHC (E), LUAD (F), PAAD (G).
