# Supplementary figures and images for: Comprehensive bioinformatics analysis reveals the prognostic value, predictive value, and immunological roles of ANLN in human cancers
Source: Front Genet. 2022 Sep 20;13:1000339. doi: 10.3389/fgene.2022.1000339 (PMC9527346; doi:10.3389/fgene.2022.1000339)

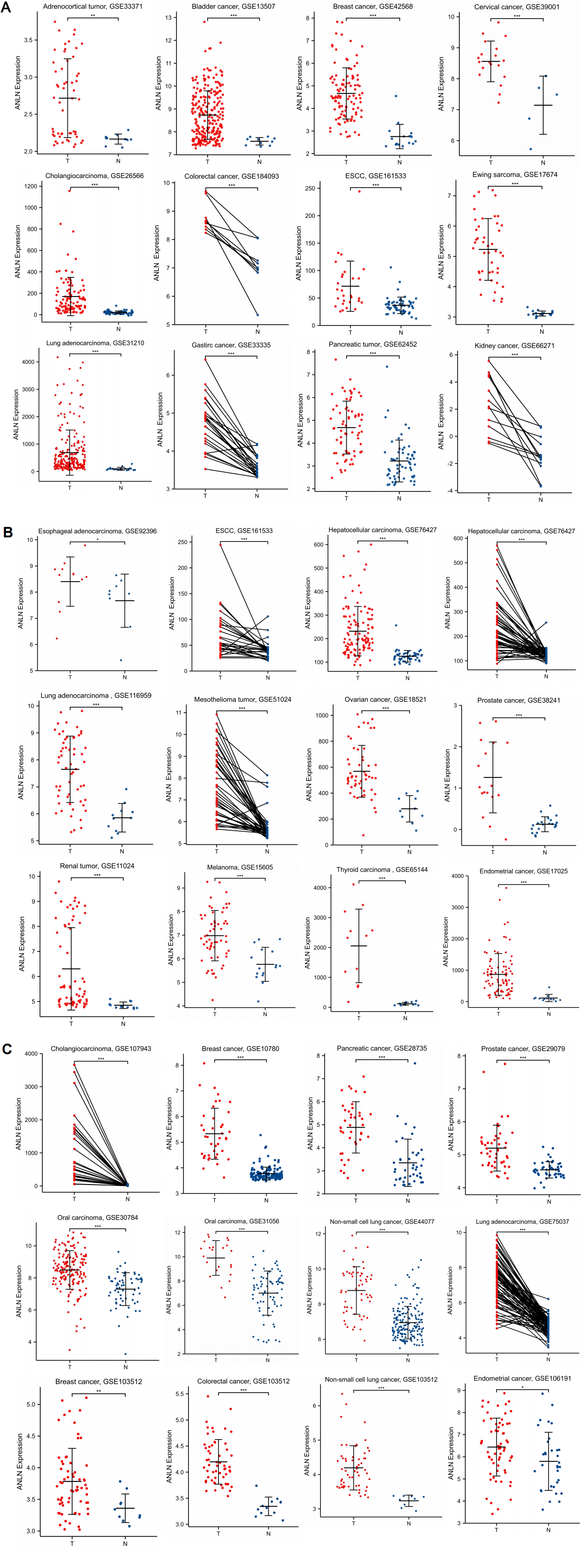

Supplement: Supplementary file 1 [file Presentation1.zip › ANLN supplementary figures/figureS1.png]

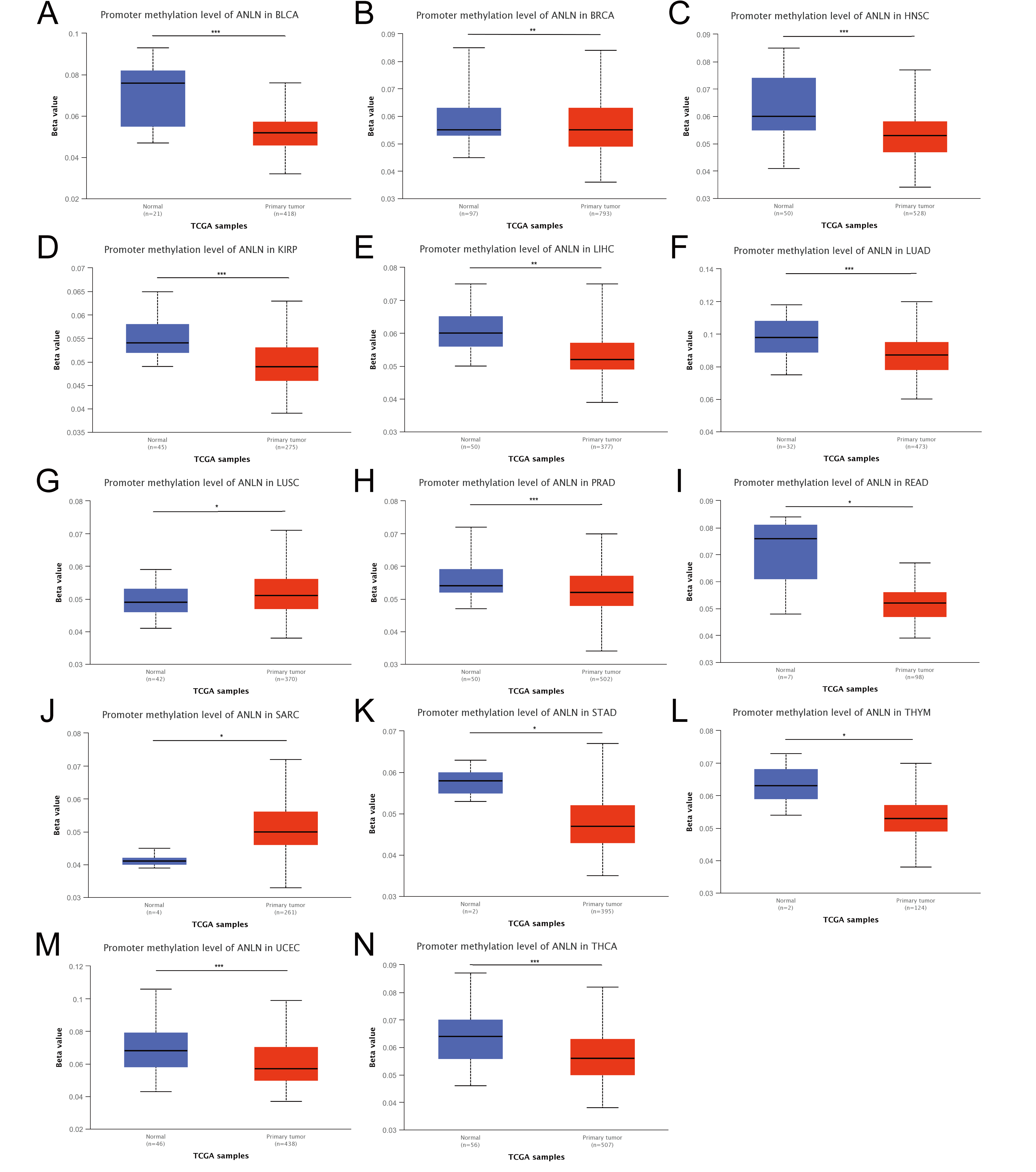

Supplement: Supplementary file 1 [file Presentation1.zip › ANLN supplementary figures/FigureS2.tif]

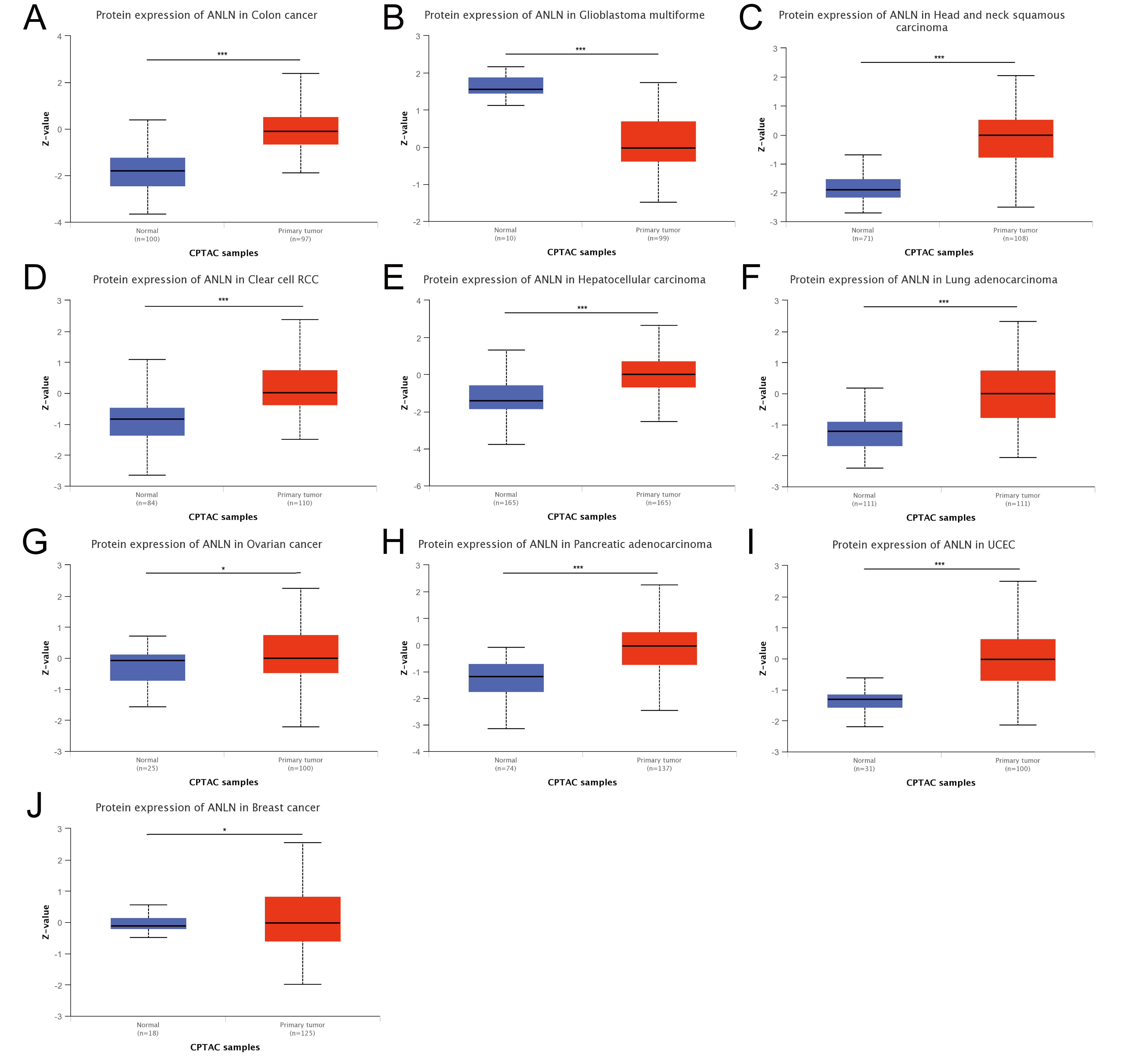

Supplement: Supplementary file 1 [file Presentation1.zip › ANLN supplementary figures/FigureS3.tif]

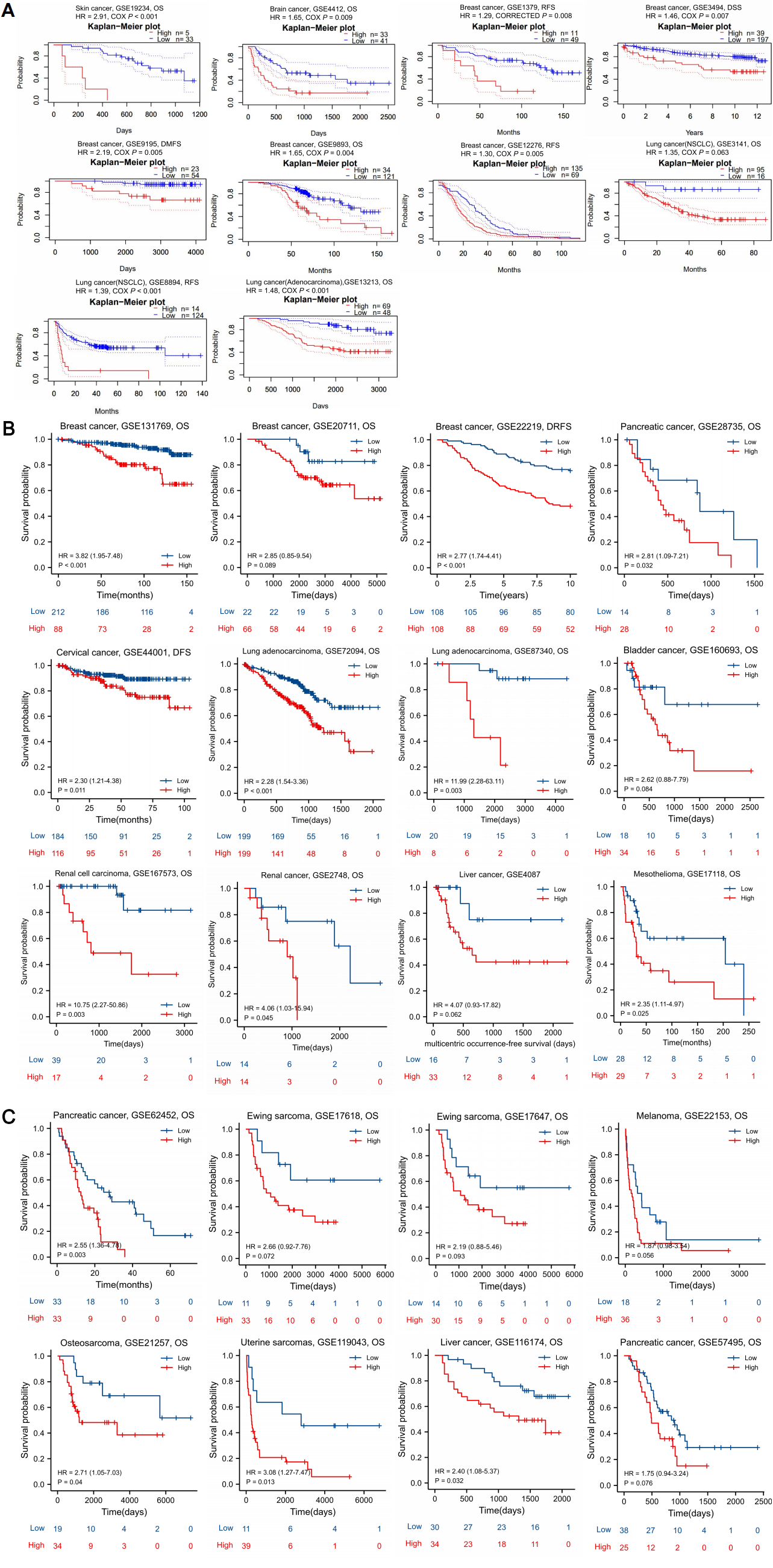

Supplement: Supplementary file 1 [file Presentation1.zip › ANLN supplementary figures/FigureS4.png]

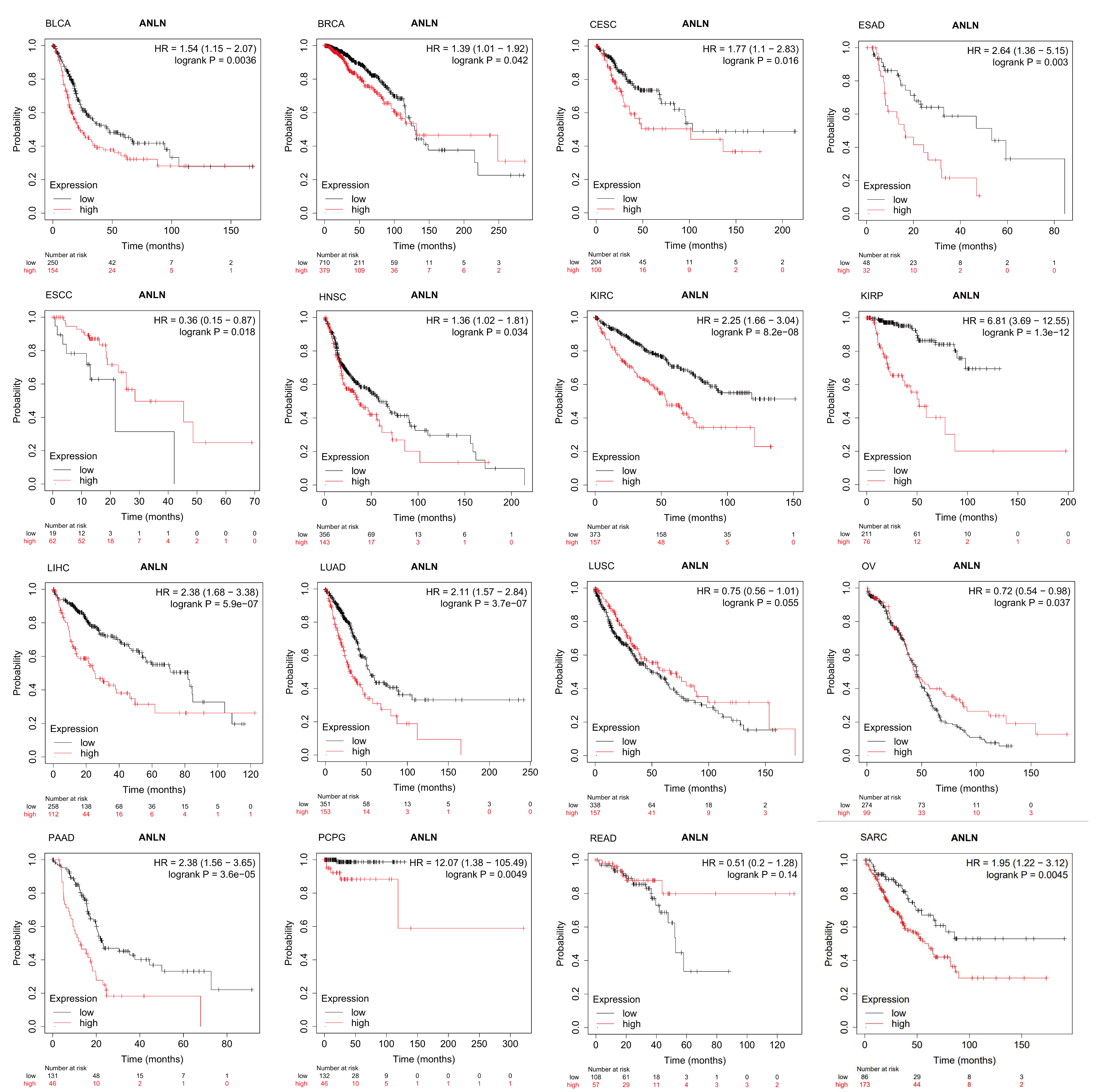

Supplement: Supplementary file 1 [file Presentation1.zip › ANLN supplementary figures/FigureS5.tif]

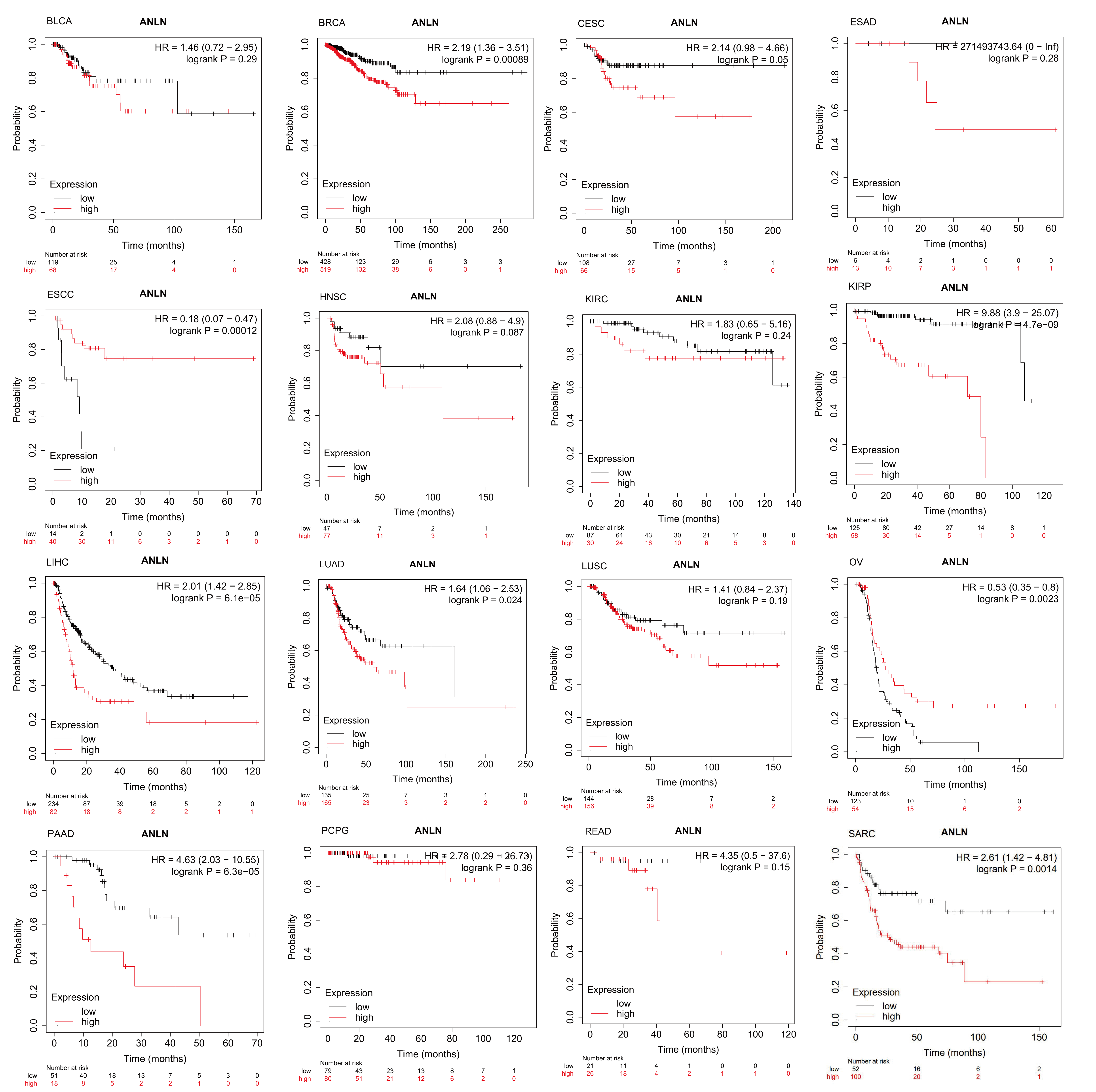

Supplement: Supplementary file 1 [file Presentation1.zip › ANLN supplementary figures/FigureS6.tif]

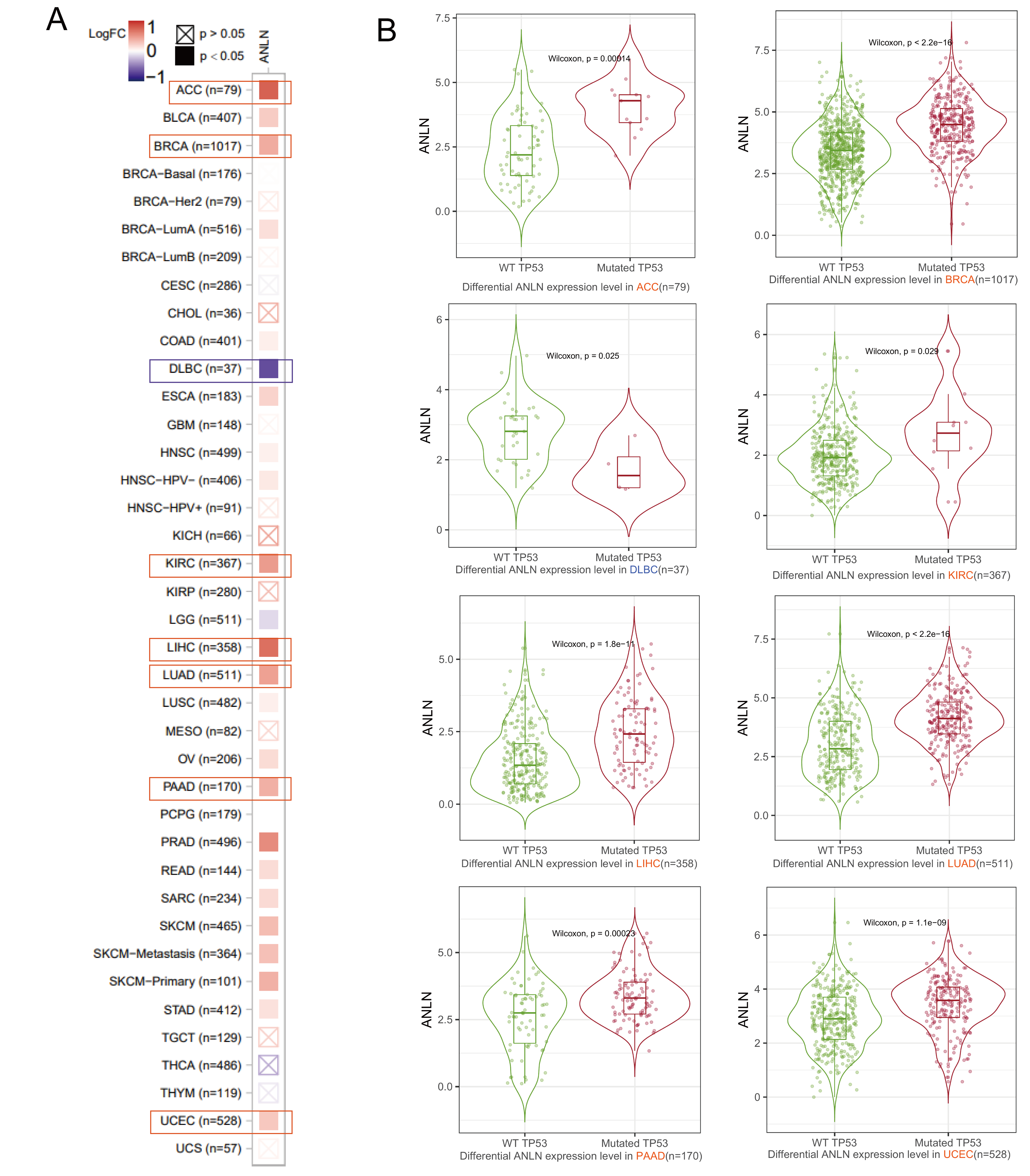

Supplement: Supplementary file 1 [file Presentation1.zip › ANLN supplementary figures/FigureS7.png]

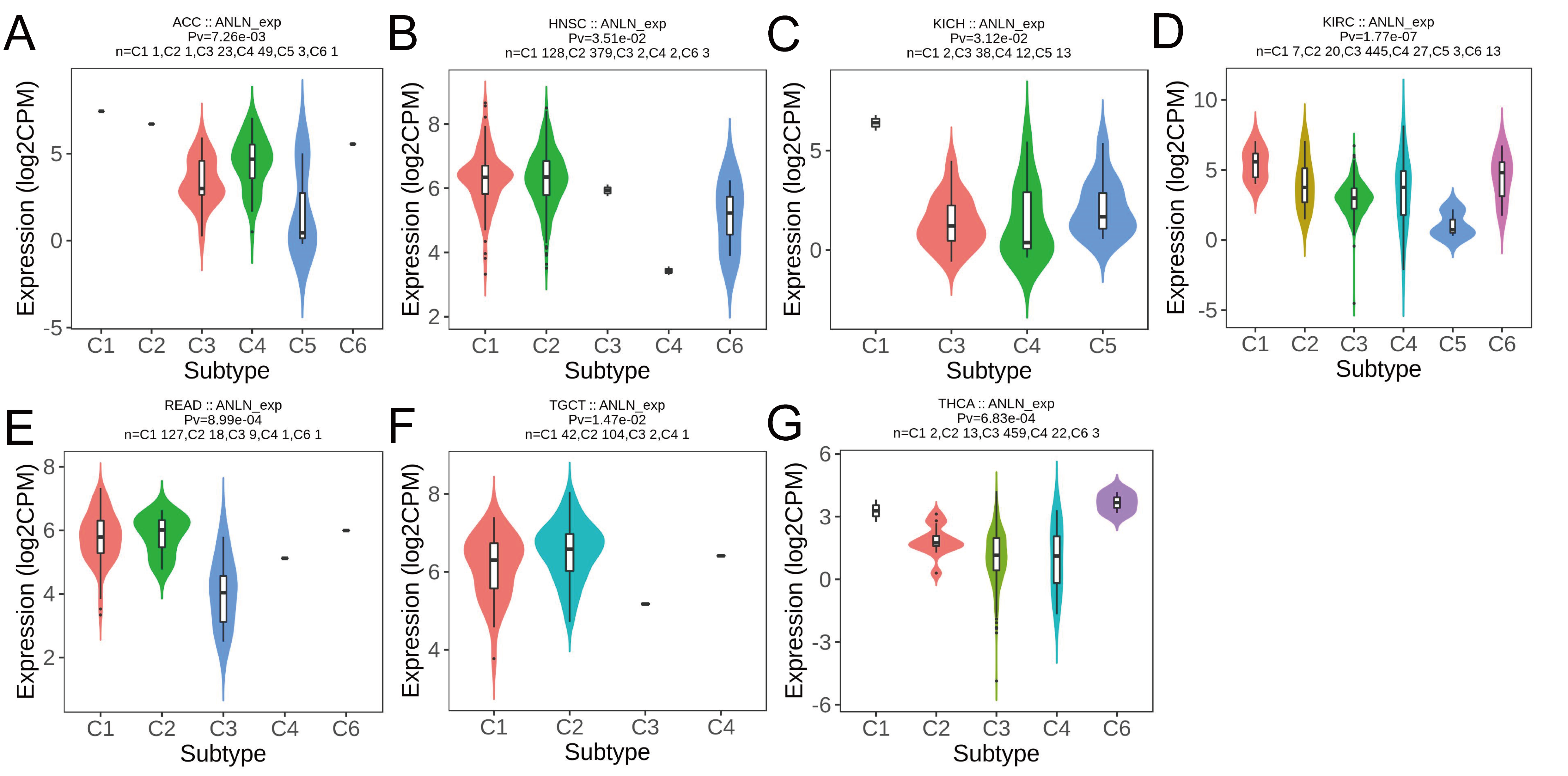

Supplement: Supplementary file 1 [file Presentation1.zip › ANLN supplementary figures/FigureS8.tif]

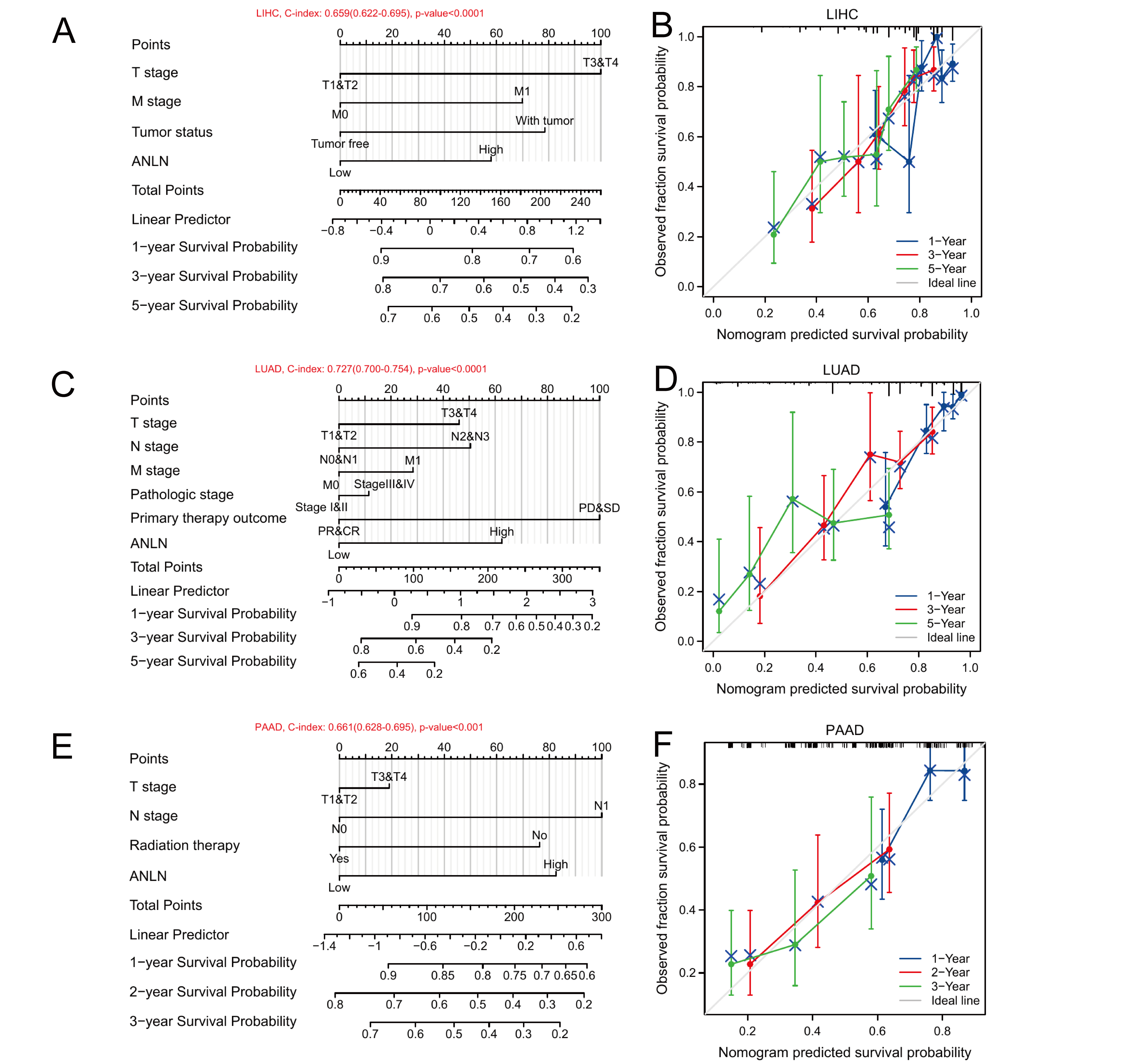

Supplement: Supplementary file 1 [file Presentation1.zip › ANLN supplementary figures/FigureS9.tif]
