# Supplementary material for: Comprehensive bioinformatics analysis reveals the prognostic value, predictive value, and immunological roles of ANLN in human cancers
Source: Front Genet. 2022 Sep 20;13:1000339. doi: 10.3389/fgene.2022.1000339 (PMC9527346; doi:10.3389/fgene.2022.1000339)
Supplement: Supplementary file 2 [file Table1.DOCX]

**Expression data (T: tumor; N: normal)**

GSE33371, adrenocortical tumor, T=55, N=10

GSE13507, bladder cancer, T=246, N=10

GSE42568, breast cancer, T=104, N=17

GSE39001, cervical cancer, T=19, N=5

GSE26566, cholangiocarcinoma, T=104, N=65

GSE184093, colorectal tumor, T=9, N=9

GSE161533, esophageal squamous cell carcinoma, unpaired: T=28, N=56; paired: T=28, N=28

GSE17674, Ewing sarcoma, T=44, N=18

GSE31210, lung adenocarcinoma, T=226, N=20

GSE33335, gastric cancer, T=25, N=25

GSE62452, pancreatic tumor, T=69, N=61

GSE66271, kidney cancer, T=13, N=13

GSE92396, esophageal adenocarcinoma, T=12, N=9

GSE76427, hepatocellular carcinoma, unpaired: T=115, N=52; paired: T=52, N=52

GSE116959, lung adenocarcinoma, T=57, N=11

GSE51024, Mesothelioma Tumor, T=42, N=42

GSE18521, ovarian cancer, T=53, N=10

GSE38241, prostate cancer, T=17, N=21

GSE11024, kidney cancer, T=67, N=12

GSE15605, melanoma, T=58, N=16

GSE65144, thyroid carcinoma, T=12, N=13

GSE17025, endometrial cancer, T=91, N=12

GSE107943, cholangiocarcinoma, T=27, N=27

GSE10780, breast cancer, T=42, N=143

GSE28735, pancreatic cancer, T=45, N=45

GSE29079, prostate cancer, T=47, N=48

GSE30784, oral carcinoma, N=167, T=62

GSE31056, oral carcinoma, N=23, T=73

GSE44077, non-small cell lung cancer, N=56, T=170

GSE75037, lung adenocarcinoma, T=83, N=83

GSE103512, breast cancer, T=65, N=10

GSE103512, colorectal cancer, T= 57, N=12

GSE103512, non-small cell lung cancer, T=60, N=9

GSE106191, endometrial cancer, T=64, N=33

**Survival data (N: patients number)**

GSE131769, breast cancer, N=300

GSE20711, breast cancer, N=88

GSE22219, breast cancer, N=216

GSE28735, pancreatic cancer, N=42

GSE44001, cervical cancer, N=300

GSE72094, lung adenocarcinoma, N=398

GSE87340, lung adenocarcinoma, N=28

GSE160693, bladder cancer, N=52

GSE167573, renal cell carcinoma, N=56

GSE2748, renal cancer, N=28

GSE40873, liver cancer, N=49

GSE17118, mesothelioma, N=57

GSE62452, pancreatic cancer, N=66

GSE17618, Ewing sarcoma, N=44

GSE17674, Ewing sarcoma, N=44

GSE22153, melanoma, N=54

GSE21257, osteosarcoma, N=53

GSE119043, uterine sarcoma, N=50

GSE116174, liver cancer, N=64

GSE57495, pancreatic cancer, N=63

**Abbreviation:**

ACC：adrenocortical carcinoma

BLCA：bladder urothelial carcinoma

BRCA：breast invasive carcinoma

CESC：cervical squamous cell carcinoma and endocervical adenocarcinoma

CHOL：cholangiocarcinoma

COAD：colon carcinoma

DLBC：lymphoid neoplasm diffuse large B-cell lymphoma

ESCA：esophageal carcinoma

GBM： glioblastoma multiforme

HNSC：Head and Neck squamous cell carcinoma

KICH：kidney chromophobe

KIRC：kidney renal clear cell carcinoma

KIRP：kidney renal papillary cell carcinoma

LAML：acute myeloid leukemia

LGG: brain lower grade glioma

LIHC: liver hepatocellular carcinoma

LUAD: lung adenocarcinoma

LUSC: lung squamous cell carcinoma

MESO: mesothelioma

OV: ovarian serous cystadenocarcinoma

OSCC: oral squamous cell carcinoma

PAAD: pancreatic adenocarcinoma

PCPG: pheochromocytoma and paraganglioma

PRAD：prostate adenocarcinoma

READ：rectum adenocarcinoma

SARC: sarcoma

SKCM: skin cutaneous melanoma

STAD: stomach adenocarcinoma

TGCT: testicular germ cell tumors

THCA: thyroid carcinoma

THYM: thymoma

UCEC: uterine corpus endometrial carcinoma

UCS: uterine carcinosarcoma

UVM: uveal melanoma
