# Supplementary material for: Comprehensive bioinformatics analysis reveals the prognostic value, predictive value, and immunological roles of ANLN in human cancers
Source: Front Genet. 2022 Sep 20;13:1000339. doi: 10.3389/fgene.2022.1000339 (PMC9527346; doi:10.3389/fgene.2022.1000339)
Supplement: Supplementary file 3 [file Table2.DOCX]

1. ACC

| Characteristics | Total(N) | Univariate analysis | |  | Multivariate analysis | |
| --- | --- | --- | --- | --- | --- | --- |
|  |  | Hazard ratio (95% CI) | P-value |  | Hazard ratio (95% CI) | P-value |
| T stage | 77 |  |  |  |  |  |
| T1&T2 | 51 | Reference |  |  |  |  |
| T3&T4 | 26 | 10.286 (3.976-26.608) | **<0.001** |  | 4.986 (1.666-14.919) | **0.004** |
| N stage | 77 |  |  |  |  |  |
| N0 | 68 | Reference |  |  |  |  |
| N1 | 9 | 2.038 (0.769-5.400) | 0.152 |  |  |  |
| M stage | 77 |  |  |  |  |  |
| M0 | 62 | Reference |  |  |  |  |
| M1 | 15 | 6.150 (2.710-13.959) | **<0.001** |  | 1.168 (0.433-3.150) | 0.760 |
| Gender | 79 |  |  |  |  |  |
| Female | 48 | Reference |  |  |  |  |
| Male | 31 | 1.001 (0.469-2.137) | 0.999 |  |  |  |
| Age | 79 |  |  |  |  |  |
| <=50 | 41 | Reference |  |  |  |  |
| >50 | 38 | 1.799 (0.846-3.824) | 0.127 |  |  |  |
| New event | 76 |  |  |  |  |  |
| No | 39 | Reference |  |  |  |  |
| Yes | 37 | 10.238 (3.050-34.359) | **<0.001** |  | 5.416 (1.555-18.857) | **0.008** |
| Radiation therapy | 76 |  |  |  |  |  |
| No | 59 | Reference |  |  |  |  |
| Yes | 17 | 1.292 (0.512-3.262) | 0.587 |  |  |  |
| ANLN | 79 |  |  |  |  |  |
| Low | 39 | Reference |  |  |  |  |
| High | 40 | 4.672 (1.973-11.063) | **<0.001** |  | 2.827 (1.063-7.517) | **0.037** |

B．BLCA

| Characteristics | Total(N) | Univariate analysis | |  | Multivariate analysis | |
| --- | --- | --- | --- | --- | --- | --- |
|  |  | Hazard ratio (95% CI) | P-value |  | Hazard ratio (95% CI) | P-value |
| T stage | 379 |  |  |  |  |  |
| T1&T2 | 124 | Reference |  |  |  |  |
| T3&T4 | 255 | 2.199 (1.515-3.193) | **<0.001** |  | 1.737 (0.872-3.461) | 0.117 |
| N stage | 369 |  |  |  |  |  |
| N0&N1 | 285 | Reference |  |  |  |  |
| N2&N3 | 84 | 2.273 (1.640-3.150) | **<0.001** |  | 1.794 (0.936-3.436) | 0.078 |
| M stage | 213 |  |  |  |  |  |
| M0 | 202 | Reference |  |  |  |  |
| M1 | 11 | 3.136 (1.503-6.544) | **0.002** |  | 0.889 (0.280-2.819) | 0.841 |
| Gender | 413 |  |  |  |  |  |
| Female | 109 | Reference |  |  |  |  |
| Male | 304 | 0.849 (0.616-1.169) | 0.316 |  |  |  |
| Age | 413 |  |  |  |  |  |
| <=70 | 233 | Reference |  |  |  |  |
| >70 | 180 | 1.421 (1.063-1.901) | **0.018** |  | 1.134 (0.681-1.887) | 0.629 |
| Radiation therapy | 387 |  |  |  |  |  |
| No | 366 | Reference |  |  |  |  |
| Yes | 21 | 0.965 (0.475-1.964) | 0.923 |  |  |  |
| Primary therapy outcome | 357 |  |  |  |  |  |
| PD&SD | 101 | Reference |  |  |  |  |
| PR&CR | 256 | 0.226 (0.162-0.315) | **<0.001** |  | 0.417 (0.235-0.739) | **0.003** |
| ANLN | 413 |  |  |  |  |  |
| Low | 207 | Reference |  |  |  |  |
| High | 206 | 1.412 (1.054-1.893) | **0.021** |  | 1.868 (1.094-3.190) | **0.022** |

C．BRCA

| Characteristics | Total(N) | Univariate analysis | |  | Multivariate analysis | |
| --- | --- | --- | --- | --- | --- | --- |
|  |  | Hazard ratio (95% CI) | P-value |  | Hazard ratio (95% CI) | P-value |
| T stage | 1079 |  |  |  |  |  |
| T1 | 276 | Reference |  |  |  |  |
| T2 | 629 | 1.334 (0.889-2.002) | 0.164 |  | 1.171 (0.737-1.858) | 0.504 |
| T3 | 139 | 1.572 (0.933-2.649) | 0.089 |  | 0.854 (0.415-1.757) | 0.668 |
| T4 | 35 | 3.755 (1.957-7.205) | **<0.001** |  | 1.600 (0.629-4.070) | 0.324 |
| N stage | 1063 |  |  |  |  |  |
| N0 | 514 | Reference |  |  |  |  |
| N1 | 357 | 1.956 (1.329-2.879) | **<0.001** |  | 1.595 (1.016-2.505) | **0.043** |
| N2 | 116 | 2.519 (1.482-4.281) | **<0.001** |  | 1.511 (0.606-3.767) | 0.376 |
| N3 | 76 | 4.188 (2.316-7.574) | **<0.001** |  | 2.101 (0.861-5.123) | 0.103 |
| M stage | 922 |  |  |  |  |  |
| M0 | 902 | Reference |  |  |  |  |
| M1 | 20 | 4.254 (2.468-7.334) | **<0.001** |  | 1.870 (0.861-4.062) | 0.114 |
| Age | 1082 |  |  |  |  |  |
| <=60 | 601 | Reference |  |  |  |  |
| >60 | 481 | 2.020 (1.465-2.784) | **<0.001** |  | 2.201 (1.521-3.184) | **<0.001** |
| Histological type | 977 |  |  |  |  |  |
| Infiltrating Ductal Carcinoma | 772 | Reference |  |  |  |  |
| Infiltrating Lobular Carcinoma | 205 | 0.827 (0.526-1.299) | 0.410 |  |  |  |
| Pathologic stage | 1059 |  |  |  |  |  |
| Stage I&Stage II | 799 | Reference |  |  |  |  |
| Stage III&Stage IV | 260 | 2.391 (1.703-3.355) | **<0.001** |  | 1.785 (0.805-3.958) | 0.154 |
| ANLN | 1082 |  |  |  |  |  |
| Low | 540 | Reference |  |  |  |  |
| High | 542 | 1.401 (1.015-1.936) | **0.041** |  | 1.580 (1.094-2.282) | **0.015** |

D．CESC

| Characteristics | Total(N) | Univariate analysis | |  | Multivariate analysis | |
| --- | --- | --- | --- | --- | --- | --- |
|  |  | Hazard ratio (95% CI) | P-value |  | Hazard ratio (95% CI) | P-value |
| T stage | 243 |  |  |  |  |  |
| T1&T2 | 212 | Reference |  |  |  |  |
| T3&T4 | 31 | 3.863 (2.072-7.201) | **<0.001** |  | 3.688 (0.713-19.078) | 0.120 |
| N stage | 195 |  |  |  |  |  |
| N0 | 134 | Reference |  |  |  |  |
| N1 | 61 | 2.844 (1.446-5.593) | **0.002** |  | 2.156 (0.627-7.417) | 0.223 |
| M stage | 127 |  |  |  |  |  |
| M0 | 116 | Reference |  |  |  |  |
| M1 | 11 | 3.555 (1.187-10.641) | **0.023** |  | 0.000 (0.000-Inf) | 0.999 |
| Radiation therapy | 306 |  |  |  |  |  |
| No | 122 | Reference |  |  |  |  |
| Yes | 184 | 1.172 (0.694-1.981) | 0.553 |  |  |  |
| Primary therapy outcome | 219 |  |  |  |  |  |
| PD&SD | 29 | Reference |  |  |  |  |
| PR&CR | 190 | 0.088 (0.048-0.160) | **<0.001** |  | 0.257 (0.052-1.276) | 0.097 |
| Histologic grade | 274 |  |  |  |  |  |
| G1&G2 | 154 | Reference |  |  |  |  |
| G3&G4 | 120 | 0.866 (0.514-1.459) | 0.589 |  |  |  |
| Histological type | 306 |  |  |  |  |  |
| Adenosquamous | 53 | Reference |  |  |  |  |
| Squamous cell carcinoma | 253 | 1.033 (0.543-1.969) | 0.920 |  |  |  |
| Age | 306 |  |  |  |  |  |
| <=50 | 188 | Reference |  |  |  |  |
| >50 | 118 | 1.289 (0.810-2.050) | 0.284 |  |  |  |
| ANLN | 306 |  |  |  |  |  |
| Low | 153 | Reference |  |  |  |  |
| High | 153 | 1.837 (1.135-2.976) | **0.013** |  | 2.922 (0.863-9.897) | 0.085 |

E. LIHC

| Characteristics | Total(N) | Univariate analysis | |  | Multivariate analysis | |
| --- | --- | --- | --- | --- | --- | --- |
|  |  | P-value | P-value |  | Hazard ratio (95% CI) | P-value |
| P-value | 370 |  |  |  |  |  |
| T1&T2 | 277 | Reference |  |  |  |  |
| T3&T4 | 93 | 2.598 (1.826-3.697) | **<0.001** |  | 2.280 (1.441-3.608) | **<0.001** |
| N stage | 258 |  |  |  |  |  |
| N0 | 254 | Reference |  |  |  |  |
| N1 | 4 | 2.029 (0.497-8.281) | 0.324 |  |  |  |
| M stage | 272 |  |  |  |  |  |
| M0 | 268 | Reference |  |  |  |  |
| M1 | 4 | 4.077 (1.281-12.973) | **0.017** |  | 1.780 (0.407-7.772) | 0.444 |
| Gender | 373 |  |  |  |  |  |
| Female | 121 | Reference |  |  |  |  |
| Male | 252 | 0.793 (0.557-1.130) | 0.200 |  |  |  |
| Age | 373 |  |  |  |  |  |
| <=60 | 177 | Reference |  |  |  |  |
| >60 | 196 | 1.205 (0.850-1.708) | 0.295 |  |  |  |
| Tumor status | 354 |  |  |  |  |  |
| Tumor free | 202 | Reference |  |  |  |  |
| With tumor | 152 | 2.317 (1.590-3.376) | **<0.001** |  | 1.910 (1.194-3.055) | **0.007** |
| ANLN | 373 |  |  |  |  |  |
| Low | 187 | Reference |  |  |  |  |
| High | 186 | 1.837 (1.293-2.609) | **<0.001** |  | 1.612 (1.017-2.553) | **0.042** |

F. LUAD

| Characteristics | Total(N) | Univariate analysis | |  | Multivariate analysis | |
| --- | --- | --- | --- | --- | --- | --- |
|  |  | Hazard ratio (95% CI) | P-value |  | Hazard ratio (95% CI) | P-value |
| T stage | 523 |  |  |  |  |  |
| T1&T2 | 457 | Reference |  |  |  |  |
| T3&T4 | 66 | 2.317 (1.591-3.375) | **<0.001** |  | 1.681 (0.938-3.014) | 0.081 |
| N stage | 510 |  |  |  |  |  |
| N0&N1 | 437 | Reference |  |  |  |  |
| N2&N3 | 73 | 2.321 (1.631-3.303) | **<0.001** |  | 1.765 (0.703-4.433) | 0.227 |
| M stage | 377 |  |  |  |  |  |
| M0 | 352 | Reference |  |  |  |  |
| M1 | 25 | 2.136 (1.248-3.653) | **0.006** |  | 1.378 (0.495-3.837) | 0.539 |
| Gender | 526 |  |  |  |  |  |
| Female | 280 | Reference |  |  |  |  |
| Male | 246 | 1.070 (0.803-1.426) | 0.642 |  |  |  |
| Age | 516 |  |  |  |  |  |
| <=65 | 255 | Reference |  |  |  |  |
| >65 | 261 | 1.223 (0.916-1.635) | 0.172 |  |  |  |
| Primary therapy outcome | 439 |  |  |  |  |  |
| PD&SD | 108 | Reference |  |  |  |  |
| PR&CR | 331 | 0.377 (0.268-0.530) | **<0.001** |  | 0.324 (0.212-0.495) | **<0.001** |
| Pathologic stage | 518 |  |  |  |  |  |
| Stage I& Stage II | 411 | Reference |  |  |  |  |
| Stage III& Stage IV | 107 | 2.664 (1.960-3.621) | **<0.001** |  | 1.137 (0.440-2.940) | 0.790 |
| ANLN | 526 |  |  |  |  |  |
| Low | 264 | Reference |  |  |  |  |
| High | 262 | 1.903 (1.420-2.550) | **<0.001** |  | 2.023 (1.337-3.059) | **<0.001** |

G. PAAD

| Characteristics | Total(N) | Univariate analysis | |  | Multivariate analysis | |
| --- | --- | --- | --- | --- | --- | --- |
|  |  | Hazard ratio (95% CI) | P-value |  | Hazard ratio (95% CI) | P-value |
| T stage | 176 |  |  |  |  |  |
| T1&T2 | 31 | Reference |  |  |  |  |
| T3&T4 | 145 | 2.023 (1.072-3.816) | **0.030** |  | 1.141 (0.584-2.229) | 0.700 |
| N stage | 173 |  |  |  |  |  |
| N0 | 50 | Reference |  |  |  |  |
| N1 | 123 | 2.154 (1.282-3.618) | **0.004** |  | 2.003 (1.109-3.619) | **0.021** |
| M stage | 84 |  |  |  |  |  |
| M0 | 79 | Reference |  |  |  |  |
| M1 | 5 | 0.756 (0.181-3.157) | 0.701 |  |  |  |
| Smoker | 144 |  |  |  |  |  |
| No | 65 | Reference |  |  |  |  |
| Yes | 79 | 1.086 (0.687-1.719) | 0.724 |  |  |  |
| Radiation therapy | 163 |  |  |  |  |  |
| No | 118 | Reference |  |  |  |  |
| Yes | 45 | 0.508 (0.298-0.866) | **0.013** |  | 0.588 (0.343-1.010) | 0.054 |
| Age | 178 |  |  |  |  |  |
| <=65 | 93 | Reference |  |  |  |  |
| >65 | 85 | 1.290 (0.854-1.948) | 0.227 |  |  |  |
| ANLN | 178 |  |  |  |  |  |
| Low | 89 | Reference |  |  |  |  |
| High | 89 | 1.993 (1.306-3.043) | **0.001** |  | 1.774 (1.124-2.798) | **0.014** |
